# A Systematic Review and Meta-Analysis Assessing the Impact of Improved Cookstove Technology Trials (ICTs) on Household Air Pollution and Human Health in Sub-Saharan Africa

**DOI:** 10.1007/s40572-025-00476-9

**Published:** 2025-01-28

**Authors:** David Dillon, Samara Reigh, Kristen M. Rappazzo, Thomas J. Luben, Anne M. Weaver

**Affiliations:** 1 Center for Public Health and Environmental Assessment, United States Environmental Protection Agency, 104 Mason Farm Rd., Chapel Hill, NC 27514 USA; 2 Department of Epidemiology and Biostatistics, University of Illinois at Chicago, Chicago, IL USA

**Keywords:** Sub-saharan Africa, Air pollution, Improved cookstove trials, Systematic review, Biomass fuels

## Abstract

**Purpose of Review:**

A major contributor to household air pollution (HAP) in sub-Saharan Africa (SSA) is unclean cooking fuel. Improved cookstove technology (ICT) interventions have been promoted as a solution, but their impacts on health are unclear. Our aim is to conduct a systematic review to explore the impacts of ICT interventions on health outcomes in SSA. We conducted a systematic review, following PRISMA guidelines, on ICT interventions in SSA from 2000-present. We performed this search in MEDLINE, PubMed, Web of Science, Web of Science CABI, and EMBASE via ProQuest. Two reviewers assessed each study using predefined inclusion/exclusion criteria and extracted data. We evaluated each study on participant selection, exposure assessment, control comparability, outcomes, analyses, and biases.

**Recent Findings:**

From 4,461 articles, k = 23 (*n* = 31,261 individuals) articles described results of ICT interventions on health outcomes. Pooled mean exposure estimates for fine particulate matter (PM_2.5_) in control and intervention groups were 102.88 µg/m^3^ (95% confidence interval [CI]I: 52.63, 153.14; I^2^ 96.9%) and 101.76 µg/m^3^ (95%CI: 57.47, 146.06; I^2^ 98.2%), respectively. Estimates for pooled mean carbon monoxide (CO) were 2.40 ppm (95% CI: 0, 8.33; I^2^ 99.0%) and 1.66 ppm (0, 4.91; I^2^ 98.5%) respectively. Of health outcomes, 19.4% were reported as significantly different between control and intervention groups.

**Summary:**

There is mixed evidence that ICT interventions influence health outcomes due to heterogeneity in study designs, sample size, stove stacking, etc. ICT interventions may decrease HAP, but other sources of air pollutant exposure are not addressed by improved cookstoves.

**Supplementary Information:**

The online version contains supplementary material available at 10.1007/s40572-025-00476-9.

## Background

Globally, air pollution contributes to the premature deaths of millions per year and costs trillions (USD) in lost economic productivity [1, 2]. The majority of these deaths occurs in low- and middle-income countries (LMICS) [3]. Many areas of the world are focused on moving towards cleaner-burning energies, with the recognition that air pollution creates a significant burden to public health [1, 4, 5]. Despite this, it is estimated that deaths attributable to air pollution will increase in the coming decades in sub-Saharan Africa (SSA) [4]. In many instances, despite increasing urbanization, many households do not have consistent access to electricity, necessitating the use of solid or biomass fuels [6, 7]. Continued reliance on household biomass/solid fuels for cooking is associated with a wide variety of diseases that impact this region of the continent [8–10]. Exposures tend to be higher in women and children, as women typically do the majority of household cooking in many areas [11].

To limit exposure to household air pollution caused by biomass fuel use, numerous trials have been conducted focused on adopting improved cookstove technologies (ICTs) to improve indoor and outdoor air quality. Despite this, it is unclear if adoption of ICTs is sustainable or if it significantly changes health outcomes. Given this landscape, it is crucial to adequately assess the extent to which ICTs impact health outcomes in SSA. The African population is expected to increase dramatically this century, with some estimates predicting a tripling of the overall population to 3.7 (95% UI: 2.48–3.84) billion people by 2100 [12]. Air pollution accounted for 1.1 million premature deaths in 2019 alone, the majority from indoor air pollution, and related economic losses reach into the billions (USD) [13]. Compounding this issue is lagging infrastructure causing approximately 530 million, as of 2014, to lack access to electricity [14]. Given this, any reduction in air pollution, particularly indoors, is important for curbing negative health impacts in populations across the subcontinent.

Previous research systematically reviewing the impacts of ICT interventions in low- and middle-income countries included six studies conducted in SSA, specifically Senegal, Rwanda, Ghana, Ethiopia, Malawi, Nigeria, and Niger [15]. Of these, only two studies reported health outcomes associated with the ICT intervention [15, 16]. Similarly, Onakomaiya et al. (2019) published a systematic review of five studies examining the impact of ICTs on blood pressure, with only two interventions in SSA [17]. Given the public health and development initiatives focused on decreasing reliance on biomass fuel it is important to understand the landscape of these interventions in SSA.

The primary objective of this study is to synthesize available scientific literature reporting the results of ICT interventions in sub-Saharan Africa. Our strategy builds on existing reviews of environmental health in SSA, as we investigate a wide range of exposures without defining health outcomes, which allows for greater inclusivity than restricting results to pulmonary or cardiovascular health. Our review also expands on previous work, with its specific focus on SSA. Providing a synthesis of this research will allow for a more complete understanding of the impacts of these interventions and the degree to which they may improve health.

## Materials and Methods

### Definitions of Air Pollution

**Fig. 1:**
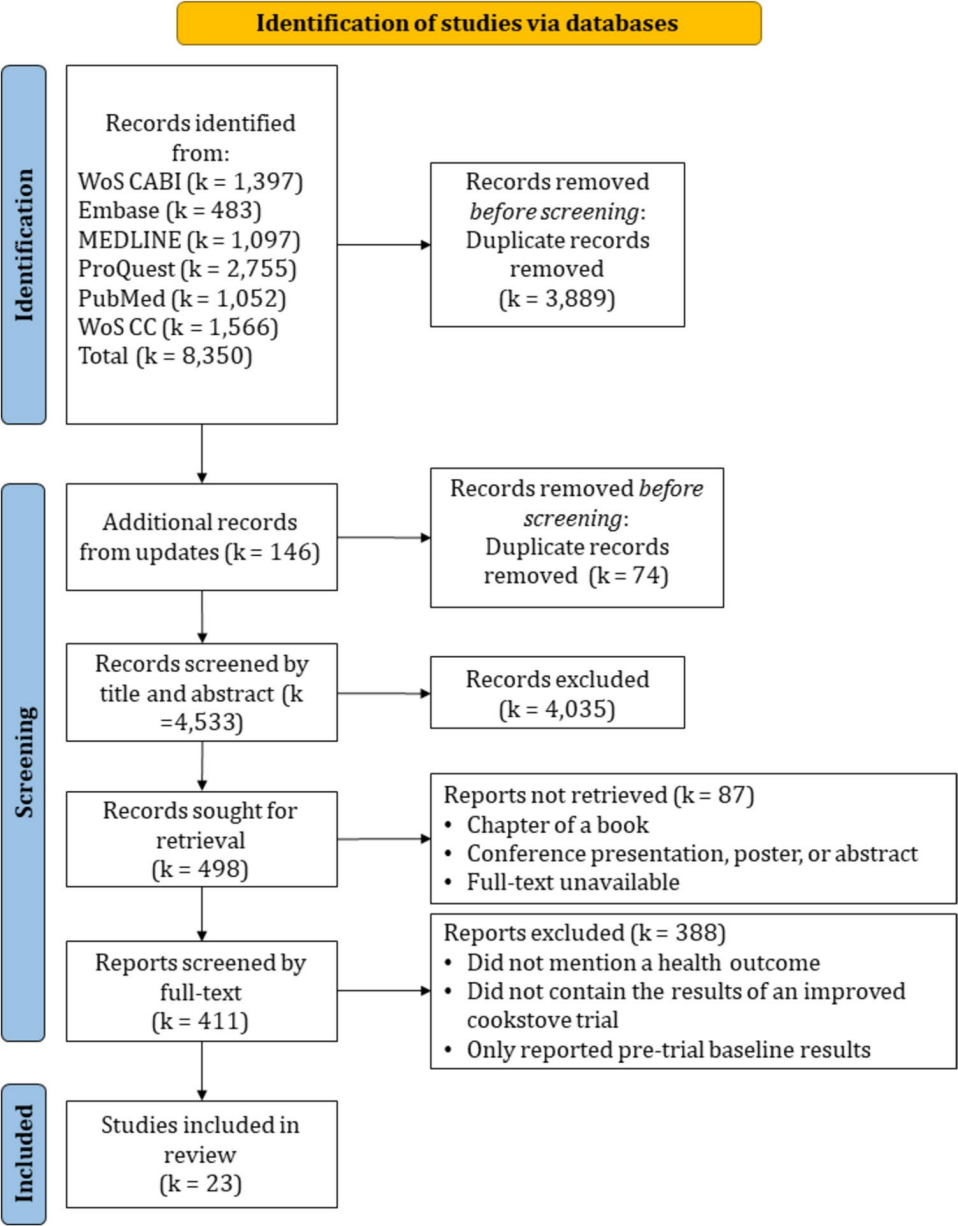
PRISMA flow diagram outlining study selection and inclusion

This review is the first in a series of studies (e.g., health impacts of ambient air pollution in sub-Saharan Africa) investigating the body of literature documenting the health impacts caused by air pollution in sub-Saharan Africa. The overarching project sought to capture all published studies examining the health impacts of air pollution in all sub-Saharan Africa. This resulted in a total of k = 411 articles, from which we focus only on ICT trials for this study, specifically the impact ICTs have had on human health outcomes. To capture as many studies as possible we included search terms for some of the most common air pollutants. These include the six United States Environmental Protection Agency’s criteria pollutants (i.e., particulate matter (PM), ozone (O_3_), nitrogen oxides (NO_X_), sulfur oxides (SO_X_), lead (Pb) and carbon monoxide (CO)) as well as other commonly studied air pollutants, such as black carbon, polycyclic aromatic hydrocarbons (PAHs), volatile organic compounds (VOCs), and semi-volatile organic compounds (SVOCs). A full list of all air pollutants included in this review appears in the search strategy (Fig. 1, Supplemental Table S1).

### Population, Exposure, Comparison, Outcome, Study Design (PECOS)

Population: Individuals of any age that reside in sub-Saharan Africa in both urban and rural settings.

Exposure: Quantitative exposures consist of reported concentrations of fine PM (PM_2.5_), coarse PM (PM_10_), O_3_, PAHs, SO_X_, NO_X_, soot, smoke, fuel use-related pollution, CO, VOCs, SVOCs, traffic-related pollution, total suspended particles, and black carbon. A full list of the term variants can be found in the search terms (supplementary table S1). Qualitative exposures (presence/absence of an unclean fuel source) were used if results were analyzed between two or more groups. Exposure to any biological airborne contaminants, such as fungal spores or bioaerosols, were excluded.

Comparator: Continued use of a traditional fuel source (e.g., charcoal, wood) or traditional stove. If the design used a single cohort longitudinally then the comparators were pre-intervention measures.

Outcome: Physiological measures that show dysfunction (with or without physician diagnosis), such as reduced lung function, DNA polymorphisms, as well as medical diagnosis of any affected organ system. Studies that reported mental health outcomes such as depression, affective disorders, sick building syndrome, etc. were included. Other biological measures of exposure, such as metabolites of PAHs, without an associated dysfunction were excluded. The only exception was studies documenting blood lead levels, as the presence of elevated lead levels constitutes a health hazard. Only human studies were included in this review, not in vitro human cell studies.

Study Design: We included randomized controlled trial, cluster-randomized trial, cross-sectional, cohort, and case-crossover studies.

### Search Strategy

Only published, peer-reviewed research written in English were considered for inclusion in this review. Records were pulled from MEDLINE; PubMed; Web of Science; Web of Science CABI; EMBASE via ProQuest dialogue. Time restrictions were January 1, 2000, to November 1, 2021. Initial inquiry did not discover any published articles before the early 1990’s. In order to gather articles from the entire subcontinent, we included every country in SSA in the search, as well as commonly used regional identifiers such as East and West Africa. In PubMed the Text Word (TW) search category was used to capture search terms that did not necessarily appear in the title or abstract. The Topic category in WoS was used and performs a similar function with the addition of Keywords Plus. Anywhere except full text (NOFT) was used in ProQuest and a similar method was used in EMBASE. No health outcomes were included in the search strategy in order to capture as many endpoints as possible. This review has been registered with PROSPERO international prospective register of systematic reviews ID: CRD42021261238 (https://www.crd.york.ac.uk/prospero/).

### Data Extraction

Studies that met the final inclusion criteria were subject to double blind extraction by authors DD, AW, SR, KR, or TL using the SWIFT-Active Screener tool [18]. Any inconsistencies in extracted data were discussed between the initial two authors, and if necessary, discussed with all authors. As many ICT trials have multiple health outcomes of interest, each of these outcomes was extracted separately. These studies were categorized in two groups reporting the results of (1) quantitative pollutant exposure or (2) qualitative exposure. If a study measured a pollutant quantitively but did not link this information to the health outcome, we treated the study as qualitative. Effect estimates for fully adjusted models were extracted; if a study only reported unadjusted estimates, this was noted.

### Quality Assessment and Risk of Bias Evaluation

All studies that met the inclusion criteria for this study were assessed for any potential risk of bias in both the study design and results using the (1) Jadad scale for the quality assessment of randomized controlled trials (2) modified Ottawa-Newcastle scales for cohort and cross-sectional (3) or an assessment tool for case-crossover studies adapted from Ding et al. 2015 [19, 20]. Each study was assessed by two authors independently and the results compared. If necessary, four (DD, AW, KR, and TL) were consulted when disagreements occurred. Studies were assessed for bias arising from the randomization process, deviations from intended interventions, missing data, outcome measurement, or in selection of the reported result. We did not assess if bias occurred as a result of participants being aware of their status within the intervention as this is inapplicable to these interventions. These studies were then categorized as good, adequate, or deficient based on final scores and rubrics (tables available in supplementary information).

#### Statistical Analysis

As exposures and outcomes were varied in this systematic review, subgroup analysis was necessary for any quantitative analyses. As such, only the same exposures and outcomes were compared in this manner. Studies with a sufficient number of comparable outcomes (≥ 3) were subject to quantitative analysis, while others were analyzed qualitatively. Given the heterogeneity in exposure measurement and reporting we perform intention-to-treat meta-analyses without exposure data to measure any impact of intervention. We report most results using the same number of significant digits the original authors report, but any results with more than three significant digits were rounded to three digits.

All statistical analyses for this meta-analysis were performed using RStudio version 4.0.3 using the packages ‘meta’, ‘metafor’, and ‘dmetar’ [16, 21, 22]. For endpoints reported by enough studies, we employed a random effects model, as populations were likely not homogenous between studies. Estimates and 95% confidence intervals were calculated. Publication bias for continuous outcomes was assessed using Egger’s, Pustejovsky-Rodgers, and Thompson regression. Publication bias for binary outcomes was assessed using Harbord and Peter’s tests. I^2^ and the p-value of Q statistics were used to evaluate heterogeneity between studies. Ƭ^2^ was estimated for binary and continuous effect size data using the Paule-Mandel method and the restricted maximum likelihood estimator when outcome data were continuous [23]. Articles occasionally reported more than one effect size per outcome, as it is common to measure more than one air pollutant or outcome at a time. More than one exposed group compared to a single control group may result in a double counting issue and raise the problem of non-independence. We dealt with this issue by only including a single outcome of one group [24]. Lastly, we performed leave-one-out sensitivity analyses to evaluate individual study influence on overall meta-estimates.

Exposures for PM_2.5_ and CO are reported differently between studies. We transformed median (IQR) and log (mean, SD) concentrations for both to mean and (SD). For median (IQR) transformations, we used the method described in Wan et al. (2014) for producing less biased estimates [25]. For studies that present log (mean, SD) we used the R package ‘fishmethods’ to back transform to mean (SD) [26]. These transformed exposures are then pooled using the metamean function in the ‘meta’ R package. For these estimates we used restricted maximum-likelihood estimator [16].

## Results

### Overall Results

As shown in the Preferred Reporting Items for Systematic Reviews and Meta-Analyses (PRISMA) flow diagram (Fig. 1), the initial search yielded 4,461 records after de-duplication. An additional 72 records were included from database alerts after the search until November 1, 2021, yielding 4,533 total records. There were 411 records included after title and abstract screening, and a total of 23 were retained following full-text screening (Table 1). The total number of individuals included in these studies was *N* = 31,261. There may be individuals included in more than one study as several of the studies identified were part of the same ICT trial. Most studies focused on respiratory health outcomes with pregnancy/birth outcomes as the second most studied topic. Following these, studies focused on cardiovascular endpoints, functional limitations, burns, eye issues, and other miscellaneous outcomes. There were four different study designs, including randomized controlled trials (including cluster), cross-sectional, case-crossover, and cohort. The studies took place in seven countries: Nigeria, Ghana, Senegal, Malawi, Kenya, Rwanda, and Ethiopia.

**Table 1:** Study characteristics

Author	Publication Year	Country	Urban/rural	Sex	Design	Intervention	Participants	Pollutant	*N*	Outcome(s)
Adane et al. [27]	2021	Ethiopia	Rural	Male & female	Cluster randomized trial	Improved biomass	Children < 5	None	5,508	Childhood acute lower respiratory infection
Alexander et al. [28]	2017	Nigeria	Urban	Female	Randomized controlled trial	Ethanol	Adults (pregnant women)	None	324	Blood pressure
Alexander et al. [29]	2018	Nigeria	Urban	Female	Randomized controlled trial	Ethanol	Adults (pregnant women)	PM_2.5_	324	Birth outcomes
Beltramo & Levine [30]	2013	Senegal	Rural	Male & female	Randomized controlled trial	Solar	Adults & Children (unspecified range)	CO	790	Respiratory and cooking-related symptoms
Burwen & Levine [31]	2012	Ghana	Rural	Female	Randomized controlled trial	Improved biomass	Adults (unspecified range)	CO	768	Respiratory outcomes and those related to smoke irritation
Critchley et al. [32]	2015	Kenya	Rural	Female	Longitudinal cohort	Improved biomass	Adults ≥ 18	VOCs	25	Spirometry and general respiratory outcomes
Dohoo et al. [33]	2012	Kenya	Rural	Female	Cross-sectional	Biogas	Adults ≥ 18	None	62	Spirometry and general respiratory outcomes
Dutta et al. [34]	2017	Nigeria	Urban	Female	Randomized controlled trial	Ethanol	Women < 18 weeks gestational age	None	77	Angiogenic factors and birth outcomes
Dutta et al. [35]	2018	Nigeria	Urban	Female	Cluster randomized trial	Ethanol	Women < 18 weeks gestational age	None	48	Indicators of hypoxia during pregnancy
Jack et al. [36]	2021	Ghana	Rural	Male & female	Cluster randomized trial	LPG	Children < 5	PM_2.5_ & CO	1,414	Birth outcomes and respiratory infections
Jary et al. [37]	2014	Malawi	Rural	Female	Randomized controlled trial	Improved biomass	Adults (unspecified range)	CO	50	Cooking-related symptoms
Kirby et al. [38]	2019	Rwanda	Rural	Male & female	Cluster randomized trial	Improved biomass	Children < 4 years	PM_2.5_	3,756	Diarrhea and respiratory infections
LaFave et al. [39]	2021	Ethiopia	Rural	Male & female	Randomized controlled trial	Improved biomass	Adults & Children (unspecified range)	PM_2.5_	1,627	Child growth, respiratory symptoms, cooking-related outcomes
Mortimer et al. [40]	2017	Malawi	Rural	Male & female	Cluster randomized trial	Improved biomass	Children < 5	None	10,543	Respiratory outcomes and general outcomes
Mortimer et al. [41]	2020	Malawi	Rural	Male & female	Cluster randomized trial	Improved biomass	Children < 5	CO	1,805	Pneumonia
Nightingale et al. [42]	2019	Malawi	Rural	Male & female	Cross-sectional	Improved biomass	Adults ≥ 18	PM_2.5_ & CO	1,481	Spirometry and general respiratory outcomes
Olopade et al. [43]	2017	Nigeria	Urban	Female	Randomized controlled trial	Ethanol	Women < 18 weeks gestational age	PM_2.5_	271	Inflammatory biomarkers
Oluwole et al. [44]	2013	Nigeria	Rural	Male & female	Cohort	Improved biomass	Women (20–60 years of age) & children (6–17 years of age)	PM_2.5_ & CO	56	Spirometry and general respiratory outcomes
Onyeneke et al. [45]	2019	Nigeria	Rural	Female	Cross-sectional	Improved biomass	Adults ≥ 18	None	400	Cooking-related symptoms
Quinn et al. [46]	2017	Ghana	Rural	Female	Randomized controlled trial	Improved biomass & LPG	Women < 28 weeks gestational age	CO	44	Blood pressure
Rylance et al. [47]	2019	Malawi	Rural	Male & female	Cross-sectional	Improved biomass	Children 6–8 years of age	CO	804	Spirometry and general respiratory outcomes
Wafula et al. [48]	2000	Kenya	Rural	Male & female	Cross-sectional	Improved biomass	Women (15–60 years of age) & children < 5 years of age	None	648	ARI and conjunctivitis
Wolff et al. [49]	2020	Rwanda	Rural	Female	Case-crossover	Improved biomass	Adults ≥ 18	None	436	Spirometry

*PM*_2.5_ fine particulate matter, *CO* carbon monoxide, *VOCs* volatile organic compounds, *ARI* acute respiratory infection, *LPG* liquefied petroleum gas

### Quality Assessment and Risk of Bias Evaluation

**Fig. 2–3:**
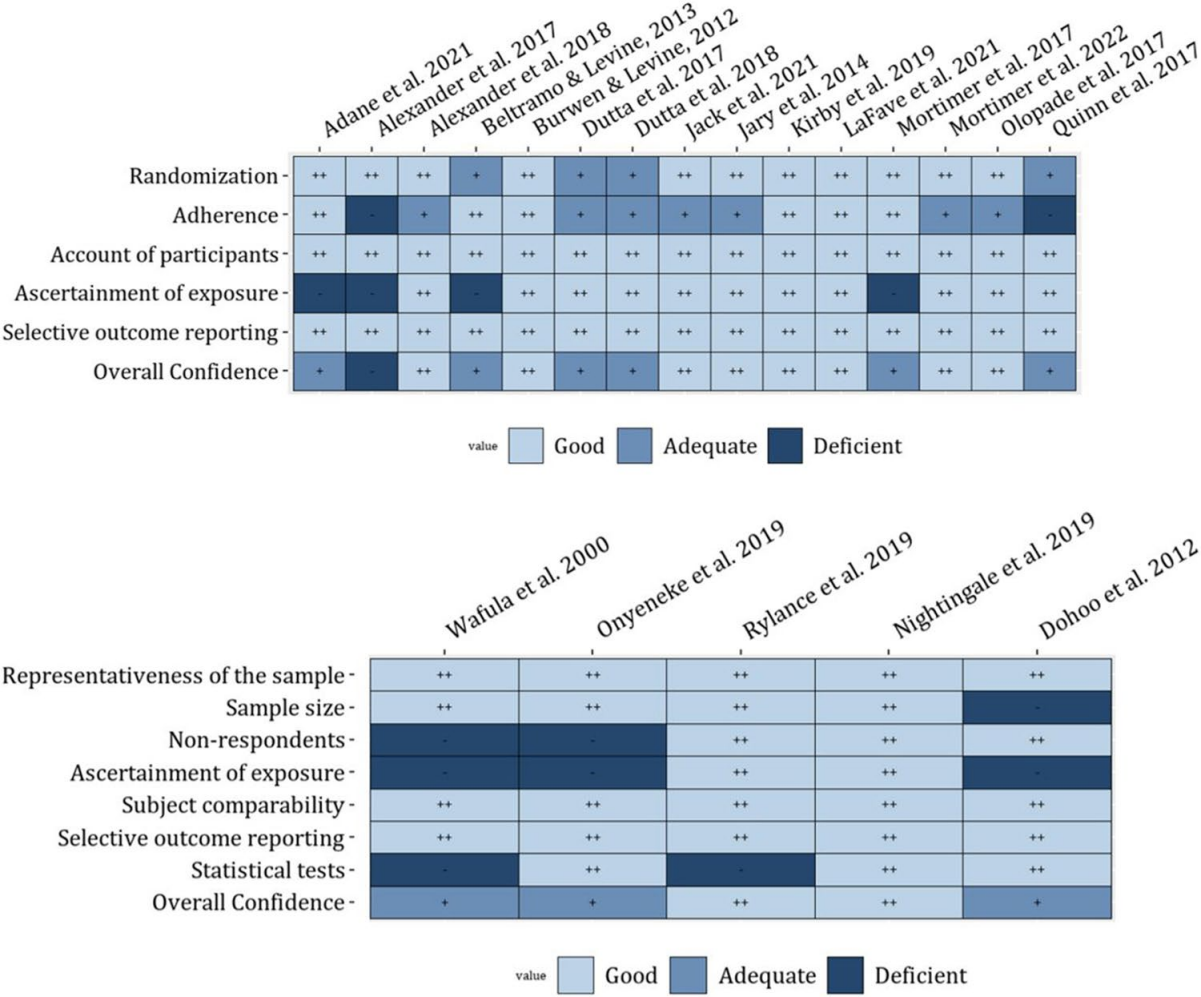
Risk of biases plots for cross-sectional and randomized controlled trials

We evaluated five cross-sectional, fifteen randomized controlled trials, two cohort, and one case-crossover studies for risk of bias (Figs. 2 and 3). Eleven of the studies were deemed low risk of bias, ten were rated as medium risk of bias, while two studies were rated as a high risk of bias. Our two cohort studies were deemed to have a low and medium risk of bias [32, 44]. Our single case-crossover study was deemed high risk of bias [49].

### Publication Bias

None of the tests showed significant publication bias, though these results should be interpreted with caution as many of the outcomes were reported by a relatively small number (i.e., three) of studies (results available in supplementary Table S2).

### Measures of Exposure

**Fig. 4:**
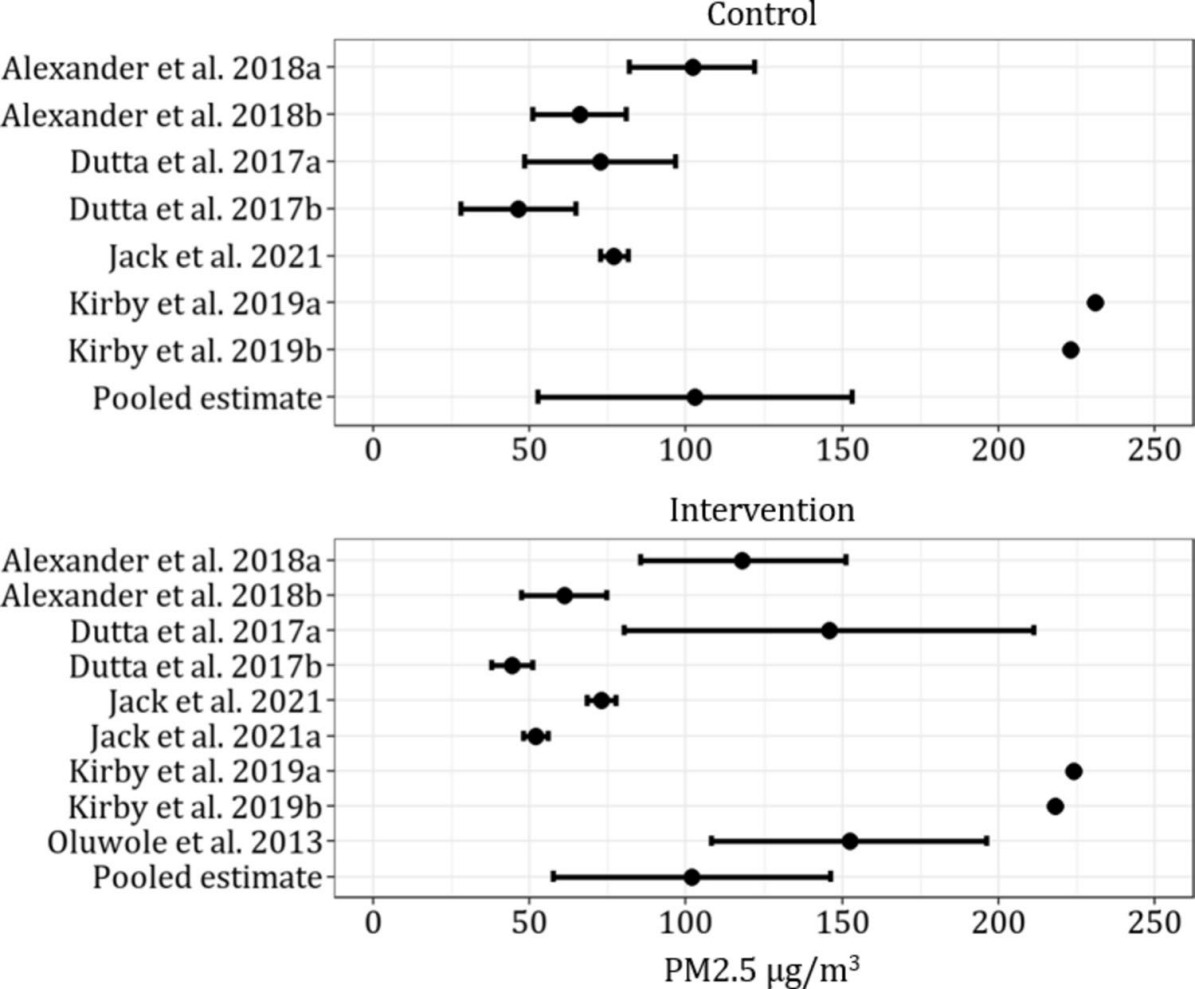
Mean (SD) reported values for PM2.5 in studies that report quantitative measures (Kirby et al. [38] did not report enough information to include error bars for measures of uncertainty)

**Fig. 5:**
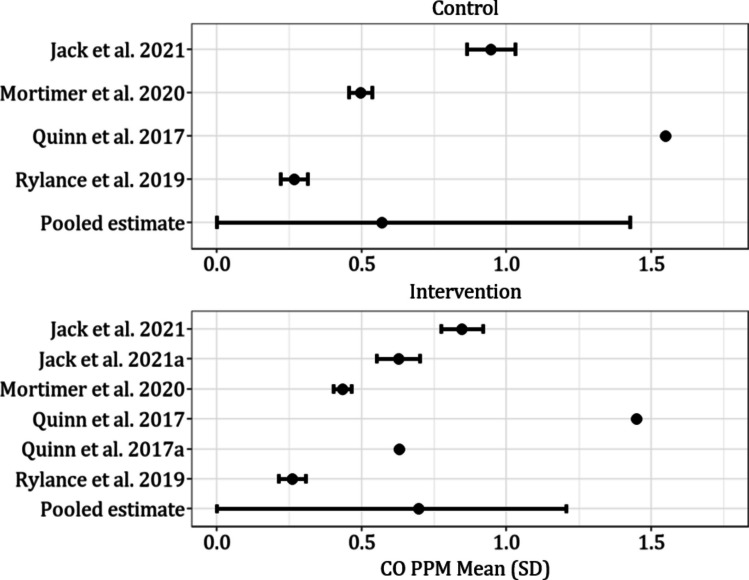
Mean (SD) reported values for CO in studies that report quantitative measures (Quinn et al. [46] did not report enough information to include error bars for measures of uncertainty)

Exposures (post-transformation) varied considerably between studies that reported quantitative measures of air pollution. In control groups the lowest and highest mean (SD) measures for PM_2.5_ were 46.3 (40.4) µg/m^3^ and 1894.2 (1980.1) µg/m^3^. In the intervention groups the lowest and highest concentrations of PM_2.5_ were 44.5 (16.2) µg/m^3^ and 152.3 (172.9) µg/m^3^. CO values showed a similarly wide range with reported concentrations ranging from 0.27 (0.33) ppm to 174.27 (91.11) ppm in the control groups and 0.26 (0.37) ppm to 17.33 (3.04) in the intervention groups. Pooled mean exposure estimates for fine particulate matter (PM_2.5_) in control and interventions groups were 102.88 µg/m^3^ (95%CI: 52.63, 153.14; I^2^ 96.9%) and 101.76 µg/m^3^ (95%CI: 57.47, 146.06; I^2^ 98.2%), respectively. Estimates for pooled mean carbon monoxide (CO) were 2.40 ppm (95% CI: 0, 8.33; I^2^ 99.0%) and 1.66 ppm (0, 4.91; I^2^ 98.5%) for control and intervention groups. Slightly more than half (12/23) of the studies reported quantitative measures of air pollution, and of these, most (7/12) explicitly integrated these measures as exposures into their models associated with health outcomes. Most studies divided the participants into control/intervention groups and focused on analyses between groups (Figs. 4 and 5).

### Geographic Distribution of Interventions

**Fig. 6:**
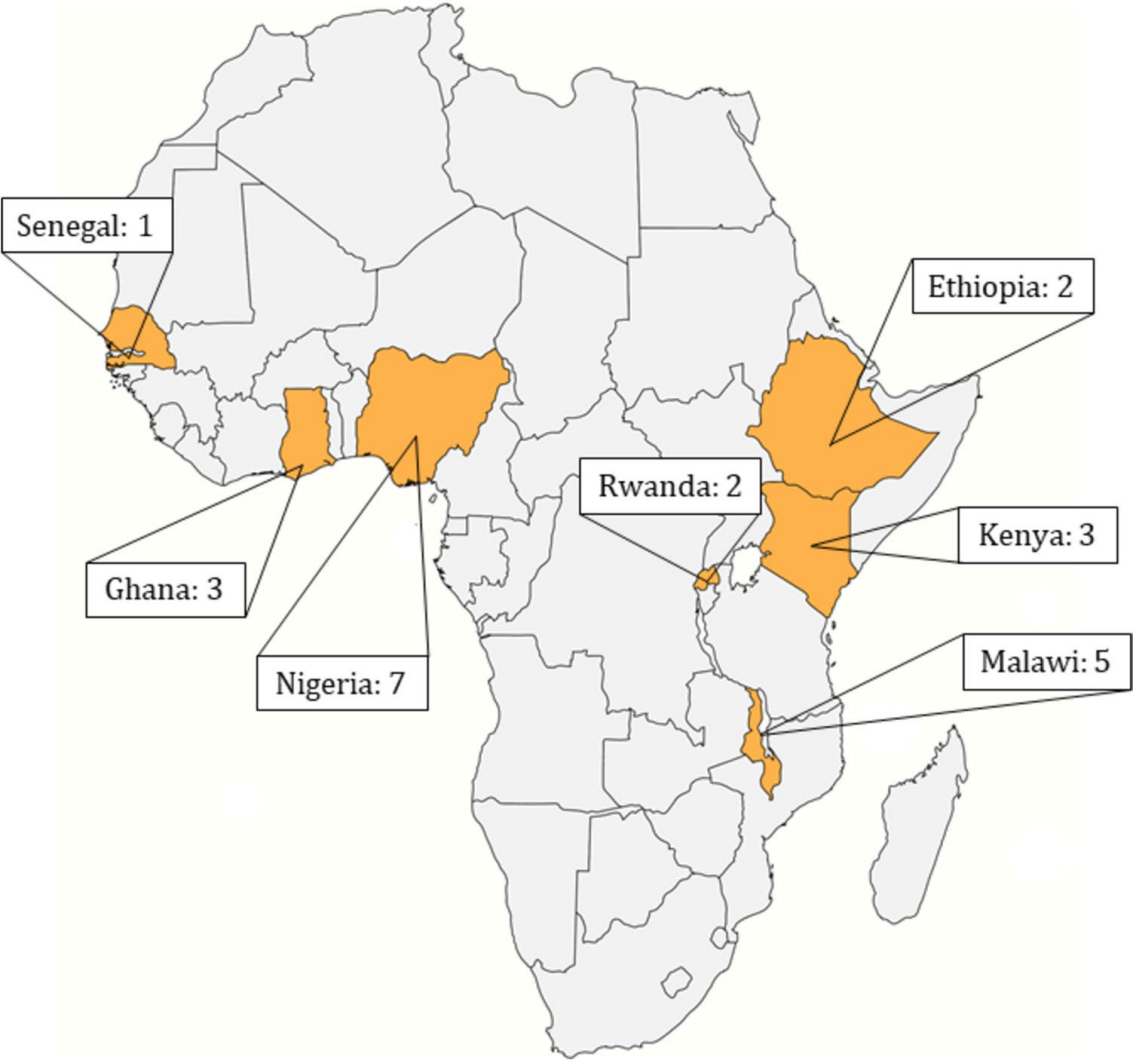
Geographic distribution of improved cookstove technology trials in sub-Saharan Africa from January 1st, 2000-November 21st, 2021

Almost half (48%) of the studies included in this review came from a few large RCTs focused on the impact of improved cookstoves. These were the Cooking and Pneumonia Study (CAPS), Ghana randomized air pollution and health study (GRAPHS), and the Household Air Pollution and Pregnancy Outcome (HAP): NCT02394574. This, in part, led to 15 of the 23 studies (65%) taking place in three countries (Fig. 6).

### Length of Time Between Intervention and Follow-Up Measures

The length of time between baseline and follow-up measurements varied between studies. Two of the RCTs focused on short-term impacts over a few weeks [37, 46]. Three studies that focused on pregnancy-related outcomes were conducted over an intermediate period from approximately < 18 weeks gestational age until birth [28, 29, 43]. The remainder of the RCT studies ranged from 6 months to 40 months of follow-up to assess the impact of the intervention [39, 30]. Lastly, six studies were cross-sectional and reported results on ongoing ICT trials (e.g., CAPS, HAP clinical trial NCT02394574), analyzed a completed trial, or were pilot studies to assess potential impacts of household air pollution [35, 42, 47, 48, 50–52].

### Results of Meta-Analyses

We calculated pooled effect estimates for spirometry measures and respiratory infections. These analyses do not include exposure data as the studies did not report results in a manner that either (1) included exposures or (2) integrated exposures into models. There were five measures of lung function commonly reported between studies: forced expiratory volume over one second (FEV_1_), forced vital capacity (FVC), FEV_1_/FVC, peak expiratory flow rate (PEFR – sometimes reported over one second as PEFR_1_), and forced expiratory flow over the middle one-half of the FVC (FEF_25−75_). For both measured and predicted results there were no significant changes in pooled estimates between control and intervention groups or pre- and post-interventions (Table 2). For the respiratory infection outcome, we combined pneumonia, acute lower respiratory infections (ALRI), and acute respiratory infections (ARI)— thus, these pooled results are for general respiratory infections. There was no difference in relative risk of respiratory infection between control and intervention groups— 0.84 (95% CI: 0.49, 1.45; I^2^: 87.5% (73.2%, 94.2%)). The results of leave one out analysis for our meta-analytic portion is available in table S3.

**Table 2:** Intention-to-treat analysis of pooled estimates for mean difference in (1) lung function measures and (2) respiratory infections between intervention and control groups

Measure	Studies (k)	Participants (*n*)	Estimate (95% CI)	I^2 ^(%)	Q (*p*-value)
Measured					
FEV_1_	4	1143	0.09 (−0.38, 0.56)	46.6 (0.0, 82.3)	5.62 (0.13)
FVC	4	619	0.036 (−0.21, 0.28)	0.00 (0.00, 84.7)	2.69 (0.44)
FEV_1_/FVC	3	195	0.13 (−0.48, 0.75)	0.0 (0.0, 89.6)	1.96 (0.38)
PEFR	3	195	0.30 (−0.93, 1.52)	69.3 (0.0, 91.0)	6.51 (0.04)
FEF_25−75_	3	195	0.15 (−0.85, 1.15)	57.6 (0.0, 87.9)	4.71 (0.09)
Predicted					
FEV_1_	4	1143	0.12 (−1.06, 1.31)	68.5 (0.0, 90.8)	6.34 (0.04)
FVC	3	195	−0.00(−0.58, 0.57)	0.00 (0.00, 89.6)	1.73 (0.42)
FEV_1_/FVC	3	195	0.14 (−0.51, 0.78)	7.9 (0.0, 90.4)	2.17 (0.34)
PEFR	3	195	0.32 (−0.93, 1.58)	70.4 (0.0, 91.3)	6.75 (0.03)
FEF_25−75_	3	195	0.07 (−0.99, 1.13)	62.5 (0.0, 89.3)	5.33 (0.07)
Measure	Studies (k)	Participants (*n*)	Relative risk (95% CI)	I^2 ^(%)	Q (*p*-value)
Respiratory infection	5	23,071	0.84 (0.49; 1.45)	87.5 (73.2, 94.2)	31.94 (< 0.01)

### Qualitative Synthesis

#### Respiratory Outcomes

The most studied respiratory outcome (k = 6) was cough [39, 37, 47, 42, 52, 31]. Five of these studies took place in rural settings, while one was set in urban Nigeria [52]. The definition of cough varied between studies, though all used self-reported data. Burwen & Levine (2012) used both number of days in the previous week with a bad cough or sore throat and presence of cough in the previous week as outcomes [31]. Others used incident cough in the previous week, questionnaires that asked, ‘do you usually have a cough when you don’t have a cold’, and in some articles it was only mentioned that participants completed interviews [31, 42, 52]. The most common way to analyze the data was to compare intervention vs. control groups without measuring environmental exposures. Control for potential confounding by covariate adjustment varied between studies as well with one study presenting only unadjusted estimates and one adjusted for clustering only [37, 31]. The remaining four studies used fully adjusted models including demographic and household information [39, 42, 47, 52]. One of the six studies used measured log_10_ CO and log_10_ PM_2.5_ as a predictor of incident cough [42]. Of these six studies one reported a significant reduction in both incident cough and number of days in the previous week experiencing coughing [31]. The other studies showed no significant differences in respiratory symptoms between control and intervention group or only an association with measured PM2.5 or CO. These results were consistent in studies that used intention-to-treat analysis in addition to observed group comparisons [39].

Lung function, as measured by spirometry, was reported in six studies [32, 42, 44, 47, 49, 50]. Four of these studies took place in rural settings, while one reported data from a Congolese refugee camp [49]. Four of these studies reported measures of lung function, including FVC, FEV_1_, ratio of FEV_1_/FVC, PEFR_1_, FEF_25−75_ [32, 44, 50, 47, 42]. Wolff et al. (2020) measured FVC, FEV_1_, and PEF_1,_ but numeric results were only given for FEV_1_ in the main manuscript (PEF_1_ results presented in figures only) [49]. The most common method of analysis was to compare control and intervention spirometry results using t-tests or an equivalent non-parametric method. One study assessed the influence of environmental exposures as a predictor of lung function [47]. Results were heterogeneous between studies, often presenting significant changes for different measures of lung function. Critchley et al. (2015) reported spirometry data collected from adult females prior to and two years after a transition to high-efficiency biomass cookstoves in rural Kenya. This study in Kenya did not report significant changes in lung function in that time period for predicted measures of lung function. They did observe significant changes for all five measures when using % predicted values and three significant changes when comparing only measured values for FEV_1_/FVC, PEFR_1_, and FEF_25−75_ [32]. Oluwole et al. (2013) measured the impact of an ICT on mothers (20 to 60 years of age) and their children (6 to 17 years of age). The only significant change post-intervention for mothers was FEF_25–75_ (L/s) and for children FEV_1_/FVC (%) [44]. Rylance et al. (2019) reported one significant difference post-intervention on FVC (L) for children 6–8 years of age. Dohoo et al. (2012) reported no significant changes to measured lung function [50]. Wolff et al. (2020) reported no significant changes in their general population but did see an increased FEV_1_ for those with preexisting airway obstruction [49].

Three studies in this review examined the association between an ICT intervention and respiratory infections [38, 48, 53]. All three studies took place in rural settings in Rwanda, Kenya, and Ethiopia. Two studies examined children under five years of age while the third examined adult women 12–60 years of age and children under five living in the same household. The three studies used different methods to assess the presence of respiratory infections. Adane et al. (2021) examined ALRIs as their outcome of interest and used Integrated Management of Childhood Illnesses (IMCI) pneumonia algorithm [53, 54]. Wafula et al. (2000) used specially-trained community-enrolled nurses to clinically evaluate participants [48]. Lastly, Kirby et al. [38] used a structured survey tool to assess presence of ARI in the seven days prior to interview as their primary outcome. Additionally, they included secondary outcomes from self-reported data including: 7-day community health worker (CHW) visit for ARI, 7-day health facility visit for ARI, 7-day ARI: DHS definition, 7-day CHW visit for ARI Demographic and Health Survey (DHS) definition, 7-day health facility visit for ARI (DHS) definition, and a visit to health facility for diarrhea within the last two months. Specifically, they defined a positive case of ARI as ‘illness with cough accompanied by rapid breathing or difficulty breathing’ [38]. Only Kirby et al. [38] measured PM_2.5_ but did not include it in models with ARI as an outcome. Adane et al. (2019) did not observe an association between improved cookstoves and a reduction in ALRI [53]. Wafula et al. (2000) reported an increased relative risk (RR) for children under five and women that used traditional fuels, with RRs of 2.6 (95% CI: 1.86, 3.63) and 2.8 (95% CI: 1.93, 4.06) respectively [48]. Lastly, Kirby et al. reported a significant reduction for 7-day ARI and 7-day CHW visit for ARI with prevalence ratios of 0.75 (95% CI: 0.60, 0.93) and 0.62 (95% CI: 0.40, 0.98) [38].

Three studies examined the impact of ICTs on pneumonia and severe pneumonia [38, 40, 55]. All took place in rural settings and were conducted in Malawi, Rwanda, and Ghana. Two of the studies examined pneumonia in children under five years of age while the study in Ghana focused on infants under 12 months. All studies used the IMCI as the criteria to indicate a case of either pneumonia or severe pneumonia. All studies report adjusted estimates derived from generalized estimating equations (GEE). Mortimer et al. (2017) reported an incidence rate ratio of 1.3 (95% CI: 0.99, 1.71) [40]. Kirby et al. reported a prevalence ratio of current pneumonia of 0.87 (95% CI: 0.58, 1.30) and 0.75 (95% CI: 0.45, 1.24) for severe pneumonia or very severe illness [38]. The third study reported on two intervention groups, one with improved biomass and the second using liquefied petroleum gas (LPG) [55]. The outcomes of this third study were additionally stratified by physician-diagnosed pneumonia/severe pneumonia and diagnoses reported by physicians and fieldworkers. In this study they compared adjusted rate ratios from GEE logistic regressions comparing improved biomass and LPG to the control group. They reported no significant differences between either intervention and the control group for either pneumonia or severe pneumonia (both physician and physician/fieldworker diagnoses). Rate ratios for physician-diagnosed cases of pneumonia were 1.08 (95% CI: 0.84–1.39) for improved biomass and 1.16 (95% CI: 0.88–1.54) for LPG. For severe pneumonia the reported rate ratios were 1.19 (95% CI: 0.74–1.91) for improved biomass and 0.98 (95% CI: 0.58–1.65) for LPG.

Wheezing as an outcome of interest for ICTs was investigated by three studies [37, 42, 47]. All three of these studies took place in rural Malawi, two of which reported on the Cooking and Pneumonia Study (CAPS) trial. All the interventions involved replacing traditional cooking methods with improved biomass cookstoves. Jary et al. (2014) did not explicitly say how they defined ‘wheeze,’ while two studies used the same prompt ‘Has your child had wheezing or whistling in the chest in the past 12 months?’ as the definition for current wheeze [37]. Jary et al. (2014) and Rylance et al. (2019) reported no significant differences in current wheeze between control and intervention groups. Nightingale et al. (2019) did not report results for current wheeze stratified by control/intervention group but did report non-significant associations between CO and wheeze with an adjusted odds ratio (OR) of 2.12 (95% CI: 0.96, 4.16).

Two studies reported asthma in children under five years of age as outcomes of interest [40, 47]. Both took place in rural Malawi and reported on the outcomes of the CAPS study. CAPS was a cluster RCT that supplied improved biomass cookstoves to replace traditional cookstoves. Mortimer et al. (2017) reported a non-significant incidence rate ratio (IRR) of 3.03 (0.51–18.11) for asthma [40]. Similarly, Rylance et al. (2019) reported an OR for severe asthma of 0.91 (0.38 to 2.19) comparing intervention to control groups [47].

Twelve studies reported respiratory outcomes that appeared only once or were reported in a way that they were not directly comparable to others in this review [29–31, 37, 39–42, 47, 49, 50, 52]. Self-reported symptoms analyzed by Burwen & Levine (2012) show a significant 7-day reduction in bad cough/sore throat, reported chest, difficulty breathing, and sore throat when not cooking [31]. Dohoo et al. (2012) saw a greater reduction in self-reported respiratory symptoms in women on biogas farms than in women on farms using traditional fuels [50]. Jary et al. (2014) reported no decrease in median exhaled CO (eCO) in their control group (0.0 ppm (IQR 3)), but a significant decrease in eCO in their intervention group (−0.5 ppm (IQR 3)) [37]. Nightingale et al. (2019) observed one significant association between any reported respiratory symptoms and CO (log_10_ ppm) with an adjusted OR of 1.46 (95% CI: 1.04–2.05), but none with PM_2.5_ (log_10_ mg/m^3^) [42]. Rylance et al. (2019) documented a reduction in COHb (%) for the intervention versus control (−0.89 (95% CI: −1.53 to − 0.26)) [47]. Lastly, Wolff et al. (2020) reported improved COPD Assessment Test (CAT) scores post-intervention compared to baseline. There was a slight improvement in scores (median CAT-score_F9/BL_ = 14 vs. 15, *p* < 0.05) for the entire intervention group nine months after baseline. Those with CAT-scores > 22 showed the greatest improvement (median CAT-score_F9/BL_ = 17 vs. 26, *p* < 0.01). None of the other 37 out of 47 results showed significant associations between an intervention and improved health outcomes.

#### Pregnancy-Related Outcomes

Three studies in this review examined size at birth as end points of interest [29, 51, 55]. One of these took place in rural Ghana and the two others in urban Nigeria, specifically Ibadan. Of note, one of these studies also included samples from African American women in Chicago as a comparison group. We will not be reporting results from the Chicago group as it is not comparable to any of the other studies included in this review. The two Nigerian studies reported the results of an RCT using ethanol cookstoves [29, 51]. The third reported on a cluster RCT with three arms consisting of a control, improved biomass, and LPG cookstoves [55]. Alexander et al. (2018) and Dutta et al. (2017) reported birthweight as a continuous outcome (in grams), while Jack et al. (2021) reported birthweight in grams, but also presented low birthweight (LBW) < 2,500 g, and small for gestational age as the < 10th percentile for gestational age [29, 51, 55]. Alexander et al. reported an estimate of 88 g greater birthweight in the ethanol stove group compared to control (95% CI: −18, 194) [29]. Similarly, Jack et al. did not report any associations with improved fuel use and birthweight with adjusted estimates of −8 g (95% CI: −81, 64) and − 15 g (95% CI: −96, 66) for biomass and LPG, respectively [55]. The third study did not provide estimates but gave the median (and range) for the control and ethanol groups which were 3000 g (2200–4000) and 3200 g (2000–4300), respectively (Dutta et al., 2017). Adjusted OR for LBW were 1.13 (95% CI: 0.86, 1.49) and 1.07 (95% CI: 0.81, 1.42) for biomass and LPG. Lastly, adjusted OR for small for gestational age were 0.61 (95% CI: 0.16, 2.24) and 1.30 (95% CI: 0.43, 3.96) for biomass and LPG.

Birth length (cm) showed an estimated effect of 0.2 cm (95% CI: −1.0, 1.4) between control and ethanol groups in Nigeria [29]. In Ghana, adjusted estimated birth length differed by 0.1 cm (95% CI: −0.9, 1.0) for improved biomass users and by −0.5 cm (95% CI: −1.6, 0.6) for LPG users [55]. Dutta et al. reported a no difference in median birth length between controls (46 cm (32–74)) and ethanol users (45.5 cm (35–52)) [51]. The final measure of size at birth was head circumference (cm). In the first study located in Nigeria there were no observed differences in the control or ethanol groups with head median circumferences of 34 (24–42) and 35 (25–44), respectively [51]. In the second Nigerian study, there was an estimated effect of 0.1 cm (95% CI: −0.7, 0.7) [29]. In Ghana, there was an estimated adjusted difference of 0.3 cm (95% CI: −0.2, 0.8) for improved biomass and 0.1 cm (95% CI: −0.3, 0.6) for LPG [55].

Two studies examined adverse birth outcomes, including miscarriage, stillbirth/neonatal mortality, perinatal mortality, preterm birth, and birth defects [29, 55]. Alexander et al. (2018) reported a risk ratio (RR) in miscarriage between ethanol and control users to be 0.4 (95% CI: 0.04, 3.1). Similarly, there was a null association between fuel use and stillbirth/neonatal death, with a RR of 0.6 (95% CI: 0.2, 1.9) [29]. Jack et al. also reported an association with neonatal mortality for improved biomass users, with an RR of 0.61 (95% CI: 0.16, 2.24), and an RR of 1.30 (95% CI: 0.43 to 3.96) for LPG users (Jack et al., 2021). Perinatal mortality for ethanol users compared to control had an RR of 0.5 (95% CI: 0.2, 1.3). The RR of preterm birth for ethanol versus control was 0.6 (95% CI: 0.3, 1.3). Alexander et al. measured differences in birth defects between the two groups, but there were none during the study period [29].

One RCT measured inflammatory biomarkers in pregnant women in urban (Ibadan) Nigeria [43]. Those in the intervention group were given an ethanol cookstove while the control continued to use biomass. The researchers measured retinol-binding protein (RBP), malondialdehyde (MDA), tumor necrosis factor-alpha (TNF-α), serum interleukin-6 (IL-6), and interleukin-8 (IL-8) as inflammatory biomarkers of interest. Baseline measures were taken at < 18 weeks gestational age (self-reported) and retaken during the third trimester (average 145 days later, range 54–187 days). Results were stratified to compare ethanol users to those who continued to use firewood and kerosene separately. There were no significant changes in biomarkers from baseline to third trimester between the intervention and kerosene groups. There was a significant difference in reduction of both TNF-α for ethanol adopters, −6.20 (SE ± 5.24), compared to those who continued to use wood, 14.03 (SE ± 5.89). The authors then used log PM_2.5_ and square root (SQRT) minutes of exposure > 100 µg/m^3^ PM_2.5_ as predictors of inflammation. Log PM_2.5_ was associated with increased levels of log IL-8 and log TNF-α, with estimates of 0.236 (95% CI: 0.025, 0.448) and 0.186 (95% CI: 0.028, 0.345), respectively. SQRT minutes exposed to > 100 µg/m^3^ PM_2.5_ was similarly associated with increased levels of log IL-8 and log TNF-α with estimates of 0.022 (95% CI: 0.003, 0.040) and 0.020 (95% CI: 0.006, 0.034) respectively. SQRT minutes was inversely associated with levels of log MDA, −0.007 (95% CI: −0.013, −0.000).

A single RCT measured chronic hypoxia in the placentas of pregnant women in urban (Ibadan) Nigeria [35]. Those in the intervention group were given an ethanol cookstove while the control continued to use biomass. The researchers assessed hypoxia using nuclear hypoxia inducible factor (HIF) expression and cytoplasmic HIF expression, both measures divided into ‘low’, ‘moderate’, and ‘high’ categories. Placental pathology was assessed by number of Hofbauer cells, number of syncytial knots, and chorionic vascular density. Results of chi-square tests show different distributions of both nuclear and cytoplasmic HIF expression. The number of Hofbauer cells, syncytial knots, and chorionic vascular density for the control group were 5.1 (SD ± 1.4), 55.6 (SD ± 7.3), and 8.8 (SD ± 1.4), respectively. The number of Hofbauer cells, syncytial knots, and chorionic vascular density for the ethanol group were 3.5 (SD ± 1.4), 41.8 (SD ± 6.9), and 6.2 (SD ± 1.6), respectively.

Dutta et al. reported results of an RCT examining associations between household air pollution and angiogenic factors in pregnant Nigerian women living in Ibadan [51]. This study measured placental growth factor (PlGF), soluble fms-like tyrosine kinase 1 (sFlt-1), placental weight, and placental ratio ((placenta weight/birthweight) × 100%). PIGF, sFlt-1, and sFlt-1/PIGF were measured in both mothers and cord blood. Controls were stratified by those who continued to use kerosene (KK) and firewood (FF). Similarly, ethanol users were stratified by previous fuel us as formerly kerosene (KE) and firewood (FE). There was one significant difference in median PIGF between KE and FE with median (Q1, Q3) measures of 118.4 (20.0, 390.6) and 38.9 (12.0, 217.0). Results on placental weight and ratio are given for the entire control and ethanol groups.

One study, Alexander et al. (2018), examined gestational age at birth as a continuous outcome between control and intervention (ethanol) groups. Those in the ethanol group gave birth an estimated one week later, 1.0 weeks (95% CI: 0.2, 1.9 weeks), than those in the control group. The authors then stratified the participants into KK, FF, KE, and FE in a similar manner to other studies in this review. Difference in mean gestational age were associated with a transition to ethanol after adjusting for both marital status and BMI, 1.6 weeks (95% CI: 0.04 to 3.2 weeks). The authors state that this difference was driven primarily by the higher rate of miscarriages and stillbirths in the FF arm of the study. Once those with adverse birth outcomes were excluded the association between fuel use and gestational age was no longer present.

#### Burns

Four studies examined the impact of improved cookstoves on accidental burns [37, 38, 40, 53]. Two studies took place in Malawi, one in Ethiopia, and one in Rwanda. All the studies reported results from rural areas, though the study located in Rwanda noted that it was ‘largely rural’ but not entirely [38]. Three of the studies reported on cluster randomized trials and one of the studies in Malawi reported on a block randomized trial [37]. Burns were reported slightly differently between studies. Two reported differences in control versus intervention group from baseline to follow-up [37, 53]. Mortimer et al. reported burns as cases per 100 child-years, while Kirby et al. collected self-reported data from the two months prior to follow-up [38, 40]. In their pilot study, Jary et al. reported differences in burns between the control and intervention groups [37]. Mortimer et al. showed a null association between improved cookstove use and burns in children with an IRR of 0.91 (95% CI: 0.37, 2.23) [40]. Adane et al. reported similar results with an IRR of 0.80 (95% CI: 0.54, 1.22) (Adane et al. 2021). In contrast, the study in Rwanda showed an association between improved cookstove use and reduction in burns in the two months prior to interview with a prevalence ratio of 0.51 (95% CI: 0.36, 0.74) [38].

#### Eye Issues

Three studies reported eye outcomes following ICT interventions [31, 37, 52]. All three took place in rural settings, in Malawi, Ghana, and Nigeria. These studies used three different study designs including RCT (Burwen & Levine, 2012), block-RCT (Jary et al. 2014), and cross-sectional (Onyeneke et al. 2019). Eye issues were reported slightly differently in each study. Jary et al. (2014) defined their outcome as ‘burning or watery eyes,’ Burwen & Levine (2012) reported ‘days with irritated eyes in the previous week,’ and Onyeneke et al. (2019) reported average yearly number of cases of eye discomfort. Jary et al. (2014) reported null associations between fuel use and burning or watery eyes. Burwen & Levine (2012) did show an association between continued use of traditional biomass fuel and number of days with eye irritation after controlling for village clustering [31]. Lastly, Onyeneke et al. (2019) provided three metrics for impact pathways. For our purposes, we used the inverse propensity score weighted average treatment effect on treated (IPSW ATT). This study reported associations between intervention and average yearly number of cases of eye discomfort with an IPSW ATT estimate of −0.40 (z-value: −0.73) [52].

#### Blood Pressure

There were two RCTs that examined the relationship between cooking fuel and a change in either systolic/diastolic blood pressure, prehypertension, or hypertension [28, 46]. One took place in rural Ghana and the other in urban Nigeria. Both studies enrolled pregnant women— Alexander et al. (2016) enrolled those between 16 and 18 weeks gestational age, while Quinn et al. [46] enrolled any women < 28 weeks gestational age. Alexander et al. followed pregnant women over six clinic visits, until 38 weeks gestational age. Quinn et al. [46] monitored blood pressure over a 72-hour period taken approximately 3–4 weeks post intervention. Alexander et al. had participants sit for ten minutes prior to blood pressure measurement, they then averaged three measures together for analysis. Quinn et al. [46] used two different protocols to measure blood pressure. The first employed a 24-hour ambulatory blood pressure monitor (ABPM) that recorded blood pressure every 20 min during the day and every 30 min at night. The second protocol, following the European Society for Hypertension guidelines, had participants take blood pressure measures for 3–4 consecutive days in the morning and evening, with at least two measures during each session. Alexander et al. (2016) presented categorical results (non-hypertensive, prehypertensive, and hypertensive) while Quinn et al. [46] reported both systolic and diastolic blood pressure as a continuous outcome. Alexander et al. did not report a different distribution of prehypertension or hypertension between control and intervention group (Alexander et al., 2016). When they stratified by previous fuel use there was a greater proportion of hypertensive participants in the group that continued to use kerosene compared to kerosene users that were provided with ethanol cookstoves. Quinn et al. [46] observed a change of −2.1 mmHg (95% CI: −6.6, 2.4) in systolic blood pressure (SBP) and − 0.1 mmHg (95% CI: −3.2, 3.0) in diastolic blood pressure (DBP) (Quinn et al. [46]).

#### Growth and Development

Two studies examined growth and development [39, 47]. These studies took place in Malawi and Ethiopia, in rural settings. Rylance et al. (2019) studied children 6–8 years of age, while LaFave et al. (2020) studied children < 36 months and children < 76 months. Both studies reported on male and female children. Rylance et al. (2019) reported effect estimates on intervention versus control for both weight-for-age z-score, height-for-age z-score, and mid-upper arm circumference (MUAC). This study reported a difference of −0.13 (SD: −0.29, 0.02) for weight-for-age z-score, −0.04 (SD: −0.20, 0.12) for height-for-age z-score, and − 0.02 (SD: −0.26, 0.21) for MUAC. LaFave et al. (2020) reported on height-for-age z-score. The results of the intention-to-treat (ITT) analysis using random effects were 0.367 (SD: 0.148) for < 36 months of age and 0.024 (SD: 0.135) for ≥ 36 months of age. LaFave et al. (2020) reported associations from random effects regression between natural log maximum PM_2.5_ levels and height-for-age z-score in children < 36 months and < 76 months. Associations were − 0.471 (SD: 0.177) for height-for-age z-score in children < 36 months and − 0.239 (0.110) for height-for-age z-score in children < 76 months.

### Daily and Functional Limitations

Two studies reported results on functional limitations [39, 42]. The studies took place in rural Malawi and Ethiopia. Nightingale et al. (2019) studied adults enrolled in the CAPS study in Malawi, while LaFave et al. (2021) studied the primary adult cook in each household. Nightingale et al. asked, ‘have your breathing problems interfered with your daily activities?’ LaFave et al. asked participants about activities of daily living (ADL) they performed and if they could (1) ‘do the activity easily’ (2) if they could ‘do it by themselves but with some difficulty; whether they needed assistance’ or (3) they ‘could not perform the task at all.’ Activities such as walking 5 km, carrying heavy loads, routine housework, etc. were included as examples [39]. Nightingale et al. reported associations between both log_10_ CO concentrations (ppm) and log_10_ PM_2.5_ concentrations (µg/m^3^) and any functional limitations. There was an adjusted OR of 1.45 (95% CI: 0.81, 2.43) for functional limitation and CO, and an adjusted OR of 0.99 (95% CI: 0.90–1.16) for functional limitation and PM_2.5_ (Nightingale et al. 2019). LaFave et al. provided two outcomes, number of ADLs with difficulty and unable to complete any ADLs. Estimates from ITT regressions were 0.19 (SE: 0.29) for number of ADLs with difficulty and 0.023 (SE: 0.04) for the inability to complete any ADLs (LaFave et al. 2021).

#### Other Outcomes

There were three studies that reported outcomes that did not fit into any of the prior categories [31, 37, 40]. Mortimer et al. reported IRRs for accidental injury, malnutrition, other, along with total number of outcomes (including asthma, which is not part of this section). The IRRs for these outcomes ranged from 0.86 to 1.32. Burwen & Levine reported on headaches in the prior week and if participants had a sick child in the prior week. The last study, Jary et al., presented results for back pain and headache. This study reported only p-values, but neither back pain nor headache differed significantly between the control and intervention group.

## Discussion

Our systematic review examines the results of ICT intervention studies completed in sub-Saharan Africa since January 1st, 2000. The results of these studies provide mixed evidence for the association between improved cookstoves and related health outcomes, with most associations being non-significant. Overall results fell into categories of (1) respiratory health (2) pregnancy- and birth-related outcomes (3) burns (4) eye/vision problems (5) blood pressure modification (6) growth and development (7) daily functional limitations, and (8) other. Overall, there was mixed evidence for reductions in indoor concentrations of both PM_2.5_ and CO following ICT interventions.

These 23 studies provide information on 252 health endpoints. Some of these endpoints were more directly comparable than others, depending on how authors both defined and measured a health outcome. Cough, for instance, relied on self-reported data in all the studies, but the definition of what constituted a positive case of cough varied between studies. These differences made it difficult to directly compare ‘cough’ as an outcome. This issue persisted across many of the other health outcomes studied. It would be advantageous to have more direct comparability between studies that examine similar health outcomes in order to (1) engage with other ICT interventions to standardize measures of interest and (2) examine previously published literature to employ similar measurements. Outcomes with higher levels of comparability were spirometry, blood pressure, etc. that have set guidelines for proper measurements [56].

Complicating these interventions, particularly in urban areas, is that there are a multitude of exposure sources not addressed by improved cookstoves that are (1) produced in the home but not caused by cooking (2) caused by others cooking with unclean fuel near another home, or (3) caused by another factor outside a household’s control. It may be that these contribute more substantially to individual exposure to air pollution—and would not be impacted by the adoption of a stove that emits lower concentrations of air pollutants. Concentrations of urban airborne PM_2.5_ in sub-Saharan African cities have been measured at concerningly high levels. In examples from West, East, and Southern Africa, PM_2.5_ levels ranged from 19 µg/m^3^ to 106 µg/m^3^ depending on conditions (wet season, dust storms, etc.) [57–59]. These examples consistently report PM levels far exceeding both the US NAAQS (annual standard: 12 µg/m^3^) and the WHO annual air quality guidelines (annual standard: 5 µg/m^3^). It may be more impactful going forward to focus international funding on initiatives to bolster national and local infrastructure, along with the adoption of national air quality standards. This would widen the scope of interventions to include not only cooking, but also waste management, regulation of vehicle emissions, industrial regulation, etc.

While improved cookstoves, if used exclusively, will reduce indoor emissions, their adoption rates and continued usage is often at lower levels than desired [11]. Continued usage is often a product of ease of use compared to traditional stoves, cooking times, how new cookstoves change the flavor of food, and the need in some cases for fuel processing pre-use. Further, an improved cookstove per house does not adequately meet household needs when multiple stoves are needed to cook, or stove-stacking is in practice. Stove-stacking occurs when there are multiple stoves in use simultaneously that may or may not use different fuel sources [60]. Shankar et al. 2020 evaluated the culture of stove-stacking on ICT programs rolled out in several countries in sub-Saharan Africa. All the studies evaluated in this review reported stove stacking occuring during their trials. This stacking is persistent for a variety of reasons, the first of which is the cost or availability of improved fuels limits their viability as a sole fuel source. Further, in many cases one stove does not provide enough energy or space to accomplish all the cooking or heating needs of a household. Many are now advocating for not the sole adoption of improved stoves but selecting the ‘cleanest stack’ [60]. However, unreliable access to electricity in many areas, particularly rural, make this impractical for some countries [61].

Many studies included in this review did not link pollutant data, making it impossible to draw conclusions about the health benefits from exposure reductions related to the cookstove intervention. Studies that did not measure pollutant exposures had no means of discovering if the improved cookstove was lowering HAP at all. While some studies utilized stove use monitors (SUMs), these are no substitute for actual air quality monitoring [29–31, 39, 43]. SUMs capture the length of cooking, but not the specific place, whether inside or outside the home, if there is stove-stacking occurring, any burning happening around the home, etc.

A third factor that may be contributing to the relatively modest associations of air pollution and health outcomes is the smaller sample sizes in many of the studies. Studies with smaller sample sizes may not be adequately powered to detect inter-group differences. Based on previously estimated guidelines for cookstove field trials, a sample size necessary to detect a 10% mean difference would require between 769 and 1,570 participants [62]. Nine studies in this review met this lower threshold while only five met the upper threshold. Given the high percentage of null results this may indicate that sample sizes were not large enough to detect changes in health outcomes.

While there is limited evidence from this systematic review that interventions significantly altered rates or prevalence of health outcomes, any reduction of indoor air pollution is an improvement, as there are causal links between air pollution and a wide range of diseases [63–68]. This is particularly salient in LMICs as the impacts of air pollution disproportionately impact these countries [1]. This includes direct impacts on health and the economic burdens associated with air pollution [13]. Future studies could strengthen evidence by using similar designs, including quantitative measures of indoor air pollution and personal exposures, monitoring of stove usage and documenting additional sources of indoor air pollution, ensuring sample sizes are large enough to capture changes in prevalence/incidence of disease, etc. This would increase comparability between studies that could strengthen the evidence base for the benefits of ICTs.

There are several limitations of this study that are important to acknowledge. The first, because of inconsistent exposure measurements/reporting between studies, we were unable to perform meta-analyses on outcomes by unit increases in either PM_2.5_ or CO. This lack of more detailed associations limits how much we are able to comment on specific reductions in air pollution corresponding to reductions in adverse health events. Potential differences in cooking practices due to different geographic and cultural settings between study sites makes direct comparisons difficult as culinary variation and preferences alters cooking time, time spent directly next to the stove, temperature of combustion, etc. These differences impact both the type and magnitude of concentrations individuals are exposed to daily. Traditional fuels also varied between studies; exposures for those that utilize charcoal vs. firewood are likely different, as firewood emits far more particulate matter (and different chemical compositions) than charcoal in both laboratory and field tests [69–71]. In addition, when control groups are continuing to be exposed to different concentrations and compositions of air pollution, the transition from a more polluting fuel to cleaner burning fuel will have different impacts on health. Further, within these intervention groups, it is likely that a transition to a much cleaner fuel (e.g., LPG) will have greater impacts on health than a transition to improved biomass. Additionally, this study may be limited by the inclusion of only articles written in English. Lastly, the lack of findings in some cases could be partially attributed to the potential reversibility or irreversibility of health outcomes during the study periods. This is further obfuscated by the, sometimes, inconsistent use of terminology when referring to respiratory diseases such as asthma and COPD [72].

A strength of this systematic review is the incorporation of four different risk of biases tools in our methods corresponding to study design. Any studies deemed to have overall high risk of bias were not included in any quantitative analyses but included for our qualitative synthesis. Our RCTs generally presented low risk of bias, apart from exposure measures, which were often presence/absence of an improved fuel source. A similar pattern held for cross-sectional and cohort studies. We excluded one study, the case-crossover from quantitative analysis, as not enough information was provided in the manuscript to ensure it was not highly biased. A second strength of this study is the scoping pool of studies examining air pollution and health in sub-Saharan Africa that these articles came from. By not limiting to one source or type of air pollutant and including all potential health outcomes, we captured a wide range of diverse studies.

As far as we are aware, this is the most comprehensive review of ICT interventions in sub-Saharan Africa. Overall, there is limited evidence that ICT interventions appreciably improve health outcomes in most reported cases. This is likely the result of numerous factors outside the domain of the interventions. First, while concentrations of household air pollutants were lowered in some cases, there are likely additional exposures outside the home from other combustion sources (e.g., vehicle emissions, industrial emissions, burning garbage). It is possible that some of these interventions would have shown significant results if there was a longer period between intervention and effect measurement, but this is sometimes not possible with field studies. However, any reduction in household air pollution is of benefit, particularly in LMICs. Quantitative exposure measurement and inclusion of such in associative models would strengthen evidence of any existing health associations. Future ICT interventions focusing on new geographic locations would increase the number of regions and culturally unique settings as well as give a better understanding of (1) stove use and adoption rates cross-culturally, (2) information on household air pollution reduction in different food cultures, and (3) provide more information on quantitative relationships between pollution reduction and health outcomes attributable to cooking methods.

## Supplementary Information

Below is the link to the electronic supplementary material.

ESM 1 (DOCX 27.7 KB)

ESM 2 (DOCX 31.8 KB)

## Data Availability

No datasets were generated or analysed during the current study.

## Declarations

### Data Sharing

The data for this study is available from the authors in the EPA ScienceHub (https://catalog.data.gov/dataset/epa-sciencehub).

### Disclaimer

The research described in this article has been reviewed by the Center for Public Health and Environmental Assessment, U.S. EPA, and approved for publication. Approval does not signify that the contents necessarily reflect the views and policies of the Agency, nor does the mention of trade names of commercial products constitute endorsement or recommendation for use.

## Abbreviations

ADL(s): Activities of daily living
ALRI(s): Acute lower respiratory infection
ARI(s): Acute respiratory infection
CAPS: Cooking and pneumonia study
CAT: COPD assessment test
CI: Confidence interval
CO: Carbon monoxide
COhB: Carboxyhemoglobin
COPD: Chronic obstructive pulmonary disease
CoV: Coefficient of variation
DHS: Demographic and Health Survey
eCO: Exhaled carbon monoxide
FEF_25-75_: Forced expiratory flow between 25% and 75% of vital capacity
FEV1 (L): Forced expiratory volume over one second
FEV1/FVC: Forced expiratory volume over one second/forced vital capacity
FVC (L): Forced vital capacity
GEE: Generalized estimating equations
HAP: Household air pollution
ICT(s): Improved cookstove technology
IRR: Incidence rate ratio
ITT: Intention-to-treat
LMICs: Low-and-middle-income countries
MUAC: Mid-upper arm circumference
O_3_: Ozone
OR: Odds ratio
PAH(s): Polycyclic aromatic hydrocarbons
PECOS: Population, Exposure, Comparison, Outcome, Study Design
PEFR: Peak expiratory flow rate
PIGF: Placental growth factor
PM_2.5_: Fine particulate matter 2.5 µm or less in diameter
PM_10_: Particulate matter 10 µm or less in diameter
PRISMA: Preferred Reporting Items for Systematic Reviews and Meta-Analyses
RCT: Randomized controlled trial
sFlt-1: Soluble fms-like tyrosine kinase 1
SSA: Sub-Saharan Africa
SQRT: Square root
SVOC(s): Semi-volatile organic compounds
UI: Uncertainty interval
VOC(s): Volatile organic compounds
