# Supplementary material for: A Systematic Review and Meta-Analysis Assessing the Impact of Improved Cookstove Technology Trials (ICTs) on Household Air Pollution and Human Health in Sub-Saharan Africa
Source: Curr Environ Health Rep. 2025 Jan 28;12(1):8. doi: 10.1007/s40572-025-00476-9 (PMC11775074; doi:10.1007/s40572-025-00476-9)
Supplement: Supplementary file 1 — (DOCX 27.7 KB) [file 40572_2025_476_MOESM1_ESM.docx]

**Supplementary information**

Table S1. List of all search terms used in the databases

| Database | Search String | Estimated Results | Notes |
| --- | --- | --- | --- |
| Web of Science | (TS=((((Africa AND (sub-Saharan OR subSaharan OR SSA OR east* OR central OR west* OR south*)) OR Angola OR Benin OR Botswana OR “Burkina Faso” OR Burundi OR “Cape Verde” OR “Cabo Verde” OR Cameroon OR “Central African Republic” OR Chad OR Comoros OR Congo OR DRC OR Djibouti OR “Cote D’Ivoire” OR “Ivory Coast” OR Eritrea OR Eswatini OR Ethiopia OR Gabon OR Gambia OR Ghana OR Guinea* OR Kenya OR Lesotho OR Liberia OR Madagascar OR Malawi OR Mali OR Maurit* OR Mozambique OR Namibia OR Niger* OR Rwanda OR “Sao Tome and Principe” OR Senegal OR Seychelles OR “Sierra Leone” OR Somalia OR Sudan OR Swaziland OR Tanzania OR Togo OR Transvaal OR Uganda OR Zambia OR Zimbabwe OR “Saint Helena” OR Bantustan OR Bophuthatswana OR Ciskei OR Kwazulu OR Lebowa OR “South African Homelands” OR “South African Republic” OR Transkei OR Venda OR Guateng OR Kwazulu-Natal OR Limpopo OR Mpumalanga OR “Northern Cape” OR “Western Cape” OR Senegal OR “Sierra Leone” OR “Western Sahara” OR Somaliland) AND (“air pollution” OR “air quality” OR (air AND (household OR ambient))) AND (PM OR PM10 OR PM2* OR PM0.1 OR “particulate matter” OR “particulate air pollutants” OR “ultrafine particle*” OR “total suspended partic*” OR “polycyclic aromatic hydrocarbon*” OR “polynuclear aromatic hydrocarbon*” OR PAH OR PAHs OR ozone OR O3 OR O-3 OR smoke OR soot OR diesel OR “black carbon” OR SO2 OR SOx OR “sulfurous anhydride” OR “sulfur dioxide” OR nitrogen* OR NO2 OR NO OR NOx OR “nitric oxide” OR “nitrogen oxide*” OR “nitrogen monoxide” OR “nitrogen dioxide” OR “carbon monoxide” OR CO OR CO2 OR “carbonic anhydride” OR “carbon dioxide” OR “volatile organic compound*” OR VOCs OR VOC OR SVOC OR “semivolatile organic compound*” OR ((traffic* OR “traffic related”) AND pollut*))))) AND DOP=(2000-2021) | 1,562 | When you run the search in WoS, it will try to change Bantustan to Baltistan. This is not the correct word.  Added more geography terms based on CABI descriptors. Mainly provinces and cities in South Africa. |
| MEDLINE | **(TS=((Africa AND (“sub-Saharan” OR “subSaharan” OR SSA OR east* OR central OR west* OR south*)) OR Angola OR Benin OR Botswana OR “Burkina Faso” OR Burundi OR “Cape Verde” OR “Cabo Verde” OR Cameroon OR “Central African Republic” OR Chad OR Comoros OR Congo OR DRC OR Djibouti OR “Cote D’Ivoire” OR “Ivory Coast” OR Eritrea OR Eswatini OR Ethiopia OR Gabon OR Gambia OR Ghana OR Guinea* OR Kenya OR Lesotho OR Liberia OR Madagascar OR Malawi OR Mali OR Maurit* OR Mozambique OR Namibia OR Niger* OR Rwanda OR “Sao Tome and Principe” OR Senegal OR Seychelles OR “Sierra Leone” OR Somalia OR Sudan OR Swaziland OR Tanzania OR Togo OR Transvaal OR Uganda OR Zambia OR Zimbabwe** OR “Saint Helena” OR Bantustan OR Bophuthatswana OR Ciskei OR Kwazulu OR Lebowa OR “South African Homelands” OR “South African Republic” OR Transkei OR Venda OR Guateng OR Kwazulu-Natal OR Limpopo OR Mpumalanga OR “Northern Cape” OR “Western Cape” OR Senegal OR “Sierra Leone” OR “Western Sahara” OR Somaliland**) OR MHX=(“Africa South of the Sahara”)) AND (TS=(“air pollution” OR “air quality” OR (air AND (household OR ambient))) OR MHX=(“Air Pollution” OR “Air Pollution, Indoor”)) AND (TS=(PM OR PM10 OR PM2* OR PM0.1 OR “particulate matter” OR “ultrafine particle*” OR “total suspended partic*” OR “polycyclic aromatic hydrocarbon*” OR “**polynuclear aromatic hydrocarbons*” **OR PAH OR PAHs OR “**carbonic anhydride” OR “carbon dioxide” OR **“carbon monoxide” OR CO OR CO2 OR ozone OR O3 OR O-3 OR smoke OR soot OR diesel OR “black carbon” OR SO2 OR SOx OR “sulfur dioxide” OR** “sulfurous anhydride” OR “particulate air pollutants” OR “nitric oxide” OR “nitrogen oxide*” OR “nitrogen monoxide” **OR “nitrogen dioxide” OR nitrogen* OR NO2 OR NO OR NOx OR “volatile organic compound*” OR VOCs OR VOC OR SVOC OR “semivolatile organic compound*” OR ((traffic* OR “traffic related”) AND pollut*)) OR MHX=(“Sulfur Oxides” OR “Sulfur Dioxide” OR Ozone OR “Particulate Matter” OR “Particle Size” OR “Air Pollution” OR “Air Pollutants” OR “Nitrogen Oxides” OR “Nitrogen Dioxide” OR “Nitric Oxide” OR “Polycyclic Aromatic Hydrocarbons” OR “Carbon Monoxide” OR “Carbon Dioxide” OR “Vehicle Emissions” OR “Volatile Organic Compounds” OR Smoke OR Soot OR Gasoline)) AND DOP=(2000-2021)** | 1,094 | When you run the search in WoS, it will try to change Bantustan to Baltistan. This is not the correct word.  Added more geography terms based on CABI descriptors. Mainly provinces and cities in South Africa.  Cannot have PM*, requires 3 characters  MeSH term “Traffic Related Pollution” not in Medline |
| CABI | **(TS=((Africa AND (“sub-Saharan” OR “subSaharan” OR SSA OR east* OR central OR west* OR south*)) OR Angola OR Benin OR Botswana OR “Burkina Faso” OR Burundi OR “Cape Verde” OR “Cabo Verde” OR Cameroon OR “Central African Republic” OR Chad OR Comoros OR Congo OR Djibouti OR “Cote D’Ivoire” OR “Ivory Coast” OR Eritrea OR Eswatini OR Ethiopia OR Gabon OR Gambia OR Ghana OR Guinea* OR Kenya OR Lesotho OR Liberia OR Madagascar OR Malawi OR Mali OR Maurit* OR Mozambique OR Namibia OR Niger* OR Rwanda OR “Sao Tome and Principe” OR Senegal OR Seychelles OR “Sierra Leone” OR Somalia OR Sudan OR Swaziland OR Tanzania OR Togo OR Transvaal OR Uganda OR Zambia OR Zimbabwe OR** “Saint Helena” OR Bantustan OR Bophuthatswana OR Ciskei OR Kwazulu OR Lebowa OR “South African Homelands” OR “South African Republic” OR Transkei OR Venda OR Guateng OR “Kwazulu-Natal” OR Limpopo OR Mpumalanga OR “Northern Cape” OR “Western Cape” OR Senegal OR “Sierra Leone” OR “Western Sahara” OR Somaliland**) OR** DE=(“Africa South of Sahara” OR “Africa South of the Sahara” OR “Central Africa” OR “North-West (South Africa)” OR “Northern Province (South Africa)” OR “South Africa” OR “Southern Africa” OR “South West Africa” OR “Sub-Saharan Africa” OR “subsaharan Africa” OR “Sub Saharan Africa” OR “West Africa” OR Benin OR “Benin(Nigeria)” OR “Benin People’s Republic” OR Angola OR Botswana OR “Burkina Faso” OR Burundi OR “Cape Verde” OR “Republic of Cape Verde” OR Cameroon OR “Central African Republic” OR Chad OR Comoros OR “Islamic Federal Republic of the Comoros” OR Congo OR “Belgian Congo” OR “Congo Democratic Republic” OR “Congo Free State” OR “Democratic Republic of the Congo” OR “Republic of the Congo” OR “Middle Congo” OR “Congo PR” OR Djibouti OR “Ivory Coast” OR “Cote d'Ivoire” OR Eritrea OR Ethiopia OR Gabon OR Gambia OR The Gambia OR Ghana OR “Equatorial Guinea” OR Guinea OR “Guinea-Bissau” OR Kenya OR Lesotho OR Liberia OR Madagascar OR Malawi OR Mali OR Mauritius OR “Republic of Mauritius” OR Mauritania OR Mozambique OR “Gaza (Mozambique)” OR Namibia OR Niger OR Nigeria OR Rwanda OR “Sao Tome and Principe” OR Somalia OR “South Sudan” OR “Southern Sudan” OR Sudan OR Swaziland OR Tanzania OR Togo OR Transvaal OR “Eastern Transvaal” OR Mpumalanga OR Uganda OR Zambia OR Zimbabwe OR “Congo Basin” OR Seychelles OR “Saint Helena” OR Bantustan OR Bophuthatswana OR Ciskei OR Kwazulu OR Lebowa OR “South African Homelands” OR “South African Republic” OR Transkei OR Venda OR Guateng OR “Kwazulu-Natal” OR Limpopo OR Mpumalanga OR “North-West (South Africa)” OR “Northern Cape” OR “Western Cape” OR “East Africa” OR Senegal OR “Sierra Leone” OR “Western Sahara”)) AND (**TS=(“air pollution” OR “air quality” OR (air AND (household OR ambient))) OR** DE=(“air pollution” OR “indoor air pollution” OR “air quality” OR “atmospheric pollution”)) AND **(TS=(PM OR PM10 OR PM2* OR PM0.1 OR “particulate matter” OR “ultrafine particle*” OR “particulate air pollutants” OR “total suspended partic*” OR “polycyclic aromatic hydrocarbon*” OR “polynuclear aromatic hydrocarbon*” OR PAH* OR PAHs OR “carbon monoxide” OR CO OR CO2 OR “carbonic anhydride” OR “carbon dioxide” OR ozone OR O3 OR O-3 OR smoke OR soot OR diesel OR “black carbon” OR SO2 OR SOx OR “sulfur dioxide” or “**sulfurous anhydride” **OR NO2 OR NO OR NOx OR nitrogen* OR “nitrogen monoxide” OR “nitrogen oxide” OR “nitrogen dioxide” OR “nitric oxide” OR “volatile organic compound*”OR “semivolatile organic compound*” OR VOCs OR VOC OR SVOC OR ((traffic* OR “traffic related”) AND pollut*)) OR** DE=(“particulate matter” OR “particle size” OR “air pollutants” OR “carbon monoxide” OR “carbon dioxide” OR ozone OR smoke OR “vehicle emissions” OR emissions OR “nitrogen oxides” OR “nitric oxide” OR “nitrous oxide” OR “sulfur dioxide” OR gasoline OR “aromatic hydrocarbons” OR “polycyclic hydrocarbons” OR “sulphur dioxide” OR gasoline)) AND DOP=(2000-2021) | 1,397 | When you run the search in WoS, it will try to change Bantustan to Baltistan. This is not the correct word.  Added more geography terms based on CABI descriptors. Mainly provinces and cities in South Africa.  Cannot have NO* in search string, need at least three characters |
| PubMed | ((("Africa"[Text Word] AND ("sub-Saharan"[Text Word] OR "subSaharan"[Text Word] OR "SSA"[Text Word] OR "east*"[Text Word] OR "central"[Text Word] OR "west*"[Text Word] OR "south*"[Text Word])) OR "Angola"[Text Word] OR "Benin"[Text Word] OR "Botswana"[Text Word] OR "Burkina Faso"[Text Word] OR "Burundi"[Text Word] OR "Cape Verde"[Text Word] OR "Cabo Verde"[Text Word] OR "Cameroon"[Text Word] OR "Central African Republic"[Text Word] OR "Chad"[Text Word] OR "Comoros"[Text Word] OR "Congo"[Text Word] OR "DRC"[Text Word] OR "Djibouti"[Text Word] OR "Cote D'Ivoire"[Text Word] OR "Ivory Coast"[Text Word] OR "Equatorial Guinea"[Text Word] OR "Eritrea"[Text Word] OR "Eswatini"[Text Word] OR "Ethiopia"[Text Word] OR "Gabon"[Text Word] OR "Gambia"[Text Word] OR "Ghana"[Text Word] OR "guinea*"[Text Word] OR "Kenya"[Text Word] OR "Lesotho"[Text Word] OR "Liberia"[Text Word] OR "Madagascar"[Text Word] OR "Malawi"[Text Word] OR "Mali"[Text Word] OR "maurit*"[Text Word] OR "Mozambique"[Text Word] OR "Namibia"[Text Word] OR "niger*"[Text Word] OR "Rwanda"[Text Word] OR "Senegal"[Text Word] OR "Seychelles"[Text Word] OR "Sierra Leone"[Text Word] OR "Somalia"[Text Word] OR "Sudan"[Text Word] OR "Swaziland"[Text Word] OR "Sao Tome and Principe"[Text Word] OR "Tanzania"[Text Word] OR "Transvaal"[Text Word] OR "Togo"[Text Word] OR "Uganda"[Text Word] OR "Zambia"[Text Word] OR "Zimbabwe"[Text Word] OR "Saint Helena"[Text Word] OR "Bantustan"[Text Word] OR "Bophuthatswana"[Text Word] OR "Ciskei"[Text Word] OR "Kwazulu"[Text Word] OR "Lebowa"[Text Word] OR "South African Homelands"[Text Word] OR "South African Republic"[Text Word] OR "Transkei"[Text Word] OR "Venda"[Text Word] OR "Guateng"[Text Word] OR "Kwazulu-Natal"[Text Word] OR "Limpopo"[Text Word] OR "Mpumalanga"[Text Word] OR "Northern Cape"[Text Word] OR "Western Cape"[Text Word] OR "Senegal"[Text Word] OR "Sierra Leone"[Text Word] OR "Western Sahara"[Text Word] OR “Somaliland”[Text Word] OR "Africa South of the Sahara"[MeSH Terms]) AND ("Air Pollution"[Text Word] OR "air quality"[Text Word] OR ("air"[Text Word] AND ("household"[Text Word] OR "ambient"[Text Word])) OR ("Air Pollution"[MeSH Terms] OR "air pollution, indoor"[MeSH Terms])) AND ("PM"[Text Word] OR "PM10"[Text Word] OR "PM2"[Text Word] OR "PM2.5"[Text Word] OR “PM0.1”[Text Word] OR "Particulate Matter"[Text Word] OR "ultrafine particle*"[Text Word] OR “total suspended partic*”[Text Word] OR "Particulate Air Pollutants"[Text Word] OR "polycyclic aromatic hydrocarbon*"[Text Word] OR "polynuclear aromatic hydrocarbon*"[Text Word] OR "PAH"[Text Word] OR "PAHs"[Text Word] OR "Carbon Monoxide"[Text Word] OR "Carbonic Anhydride"[Text Word] OR "Carbon Dioxide"[Text Word] OR "CO"[Text Word] OR "CO2"[All Fields] OR "Ozone"[Text Word] OR "O3"[Text Word] OR "O-3"[Text Word] OR "smoke"[Text Word] OR "diesel"[Text Word] OR "black carbon"[Text Word] OR "soot"[Text Word] OR "SO2"[Text Word] OR "SOx"[Text Word] OR "Sulfur Dioxide"[Text Word] OR "Sulfurous Anhydride"[Text Word] OR "nitrogen*"[Text Word] OR "NO"[Text Word] OR "NOx"[Text Word] OR "NO2"[Text Word] OR "nitrogen dioxide"[Text Word] OR "Nitric Oxide"[Text Word] OR "nitrogen oxide*"[Text Word] OR "Nitrogen Monoxide"[Text Word] OR "volatile organic compound*"[Text Word] OR "VOC"[Text Word] OR "VOCs"[Text Word] OR "SVOC"[Text Word] OR "semivolatile organic compound*"[Text Word] OR ("traffic*"[Text Word] AND "pollution"[Text Word]) OR "Traffic-Related Pollution"[Text Word] OR ("Sulfur Oxides"[MeSH Terms] OR "Sulfur Dioxide"[MeSH Terms] OR "Ozone"[MeSH Terms] OR "Particulate Matter"[MeSH Terms] OR "Particle Size"[MeSH Terms] OR "Air Pollution"[MeSH Terms] OR "Air Pollutants"[MeSH Terms] OR "Nitrogen Oxides"[MeSH Terms] OR "Polycyclic Aromatic Hydrocarbons"[MeSH Terms] OR "Carbon Monoxide"[MeSH Terms] OR "Carbon Dioxide"[MeSH Terms] OR "Nitric Oxide"[MeSH Terms] OR "Traffic-Related Pollution"[MeSH Terms] OR "soot"[MeSH Terms] OR "gasoline"[MeSH Terms] OR "smoke"[MeSH Terms] OR "Vehicle Emissions"[MeSH Terms] OR "Volatile Organic Compounds"[MeSH Terms]))) AND (2000:2021[pdat]) | 1,050 | Cannot search for PM*, PM1*, or PM2*. Need at least four characters.  Added more geography terms based on CABI descriptors. Mainly provinces and cities in South Africa |
| ProQuest | ab,ti(((Africa AND (sub-Saharan OR subSaharan OR SSA OR east* OR central OR west* OR south*)) OR Angola OR Benin OR Botswana OR “Burkina Faso” OR Burundi OR “Cape Verde” OR “Cabo Verde” OR Cameroon OR “Central African Republic” OR Chad OR Comoros OR Congo OR DRC OR Djibouti OR “Cote D’Ivoire” OR “Ivory Coast” OR Eritrea OR Eswatini OR Ethiopia OR Gabon OR Gambia OR Ghana OR Guinea* OR Kenya OR Lesotho OR Liberia OR Madagascar OR Malawi OR Mali OR Maurit* OR Mozambique OR Namibia OR Niger* OR Rwanda OR “Sao Tome and Principe” OR Senegal OR Seychelles OR “Sierra Leone” OR Somalia OR Sudan OR Swaziland OR Tanzania OR Togo OR Transvaal OR Uganda OR Zambia OR Zimbabwe OR “Saint Helena” OR Bantustan OR Bophuthatswana OR Ciskei OR Kwazulu OR Lebowa OR “South African Homelands” OR “South African Republic” OR Transkei OR Venda OR Guateng OR “Kwazulu-Natal” OR Limpopo OR Mpumalanga OR “Northern Cape” OR “Western Cape” OR Somaliland) AND (“air pollution” OR “air quality” OR (air AND (household OR ambient))) AND (PM* OR PM1* OR PM2* OR PM0.1 OR “particulate matter” OR “particulate air pollutants” OR “ultrafine particle*” OR “total suspended partic*” OR “polycyclic aromatic hydrocarbon*” OR PAH OR PAHs OR “carbon monoxide” OR CO OR CO2 OR “carbonic anhydride” OR ozone OR O3 OR O-3 OR smoke OR soot OR diesel OR “black carbon” OR SO2 OR SOx OR “sulfur dioxide” OR nitrogen OR NO2 OR NO OR NOx OR “nitrogen dioxide” OR “Sulfurous Anhydride” OR “Particulate Air Pollutants” OR “Polynuclear Aromatic Hydrocarbon*” OR “Carbonic Anhydride” OR “Carbon Dioxide” OR “Nitric Oxide” OR “Nitrogen Oxide*” OR “Nitrogen Monoxide” OR “volatile organic compound*” OR VOCs OR VOC OR “SVOC” OR “semivolatile organic compound*” OR ((traffic* OR “traffic related”) AND pollut*))) AND PD(2000-2021) | 1,415 | When searching ProQuest, you may notice that you receive a different number of results each time you search or the number of results at the top of your search page is not accurate. To see the true number of results, you will need to scroll to the end of the results page.  Added more geography terms based on CABI descriptors. Mainly provinces and cities in South Africa |
| EMBASE | ab,su,ti(((Africa AND (sub-Saharan OR subSaharan OR SSA OR east* OR central OR west* OR south*)) OR Angola OR Benin OR Botswana OR “Burkina Faso” OR Burundi OR “Cape Verde” OR “Cabo Verde” OR Cameroon OR “Central African Republic” OR Chad OR Comoros OR Congo OR DRC OR Djibouti OR “Cote D’Ivoire” OR “Ivory Coast” OR Eritrea OR Eswatini OR Ethiopia OR Gabon OR Gambia OR Ghana OR Guinea* OR Kenya OR Lesotho OR Liberia OR Madagascar OR Malawi OR Mali OR Maurit* OR Mozambique OR Namibia OR Niger* OR Rwanda OR “Sao Tome and Principe” OR Senegal OR Seychelles OR “Sierra Leone” OR Somalia OR Sudan OR Swaziland OR Tanzania OR Togo OR Transvaal OR Uganda OR Zambia OR Zimbabwe OR “Saint Helena” OR Bantustan OR Bophuthatswana OR Ciskei OR Kwazulu OR Lebowa OR “South African Homelands” OR “South African Republic” OR Transkei OR Venda OR Guateng OR “Kwazulu-Natal” OR Limpopo OR Mpumalanga OR “Northern Cape” OR “Western Cape” OR Somaliland OR EMB.EXACT.EXPLODE("Africa south of the Sahara")) AND (“air pollution” OR “air quality” OR (air AND (household OR ambient)) OR EMB.EXACT.EXPLODE("air pollution") OR EMB.EXACT.EXPLODE("air quality") OR EMB.EXACT.EXPLODE("ambient air") OR EMB.EXACT.EXPLODE("indoor air pollution")) AND (PM1* OR PM2* OR PM0.1 OR “particulate matter” OR “ultrafine particle*” OR “particulate air pollutants” OR “total suspended partic*” OR “polycyclic aromatic hydrocarbon*” OR “polynuclear aromatic hydrocarbon*” OR PAH OR PAHs OR “carbon monoxide” OR CO OR CO2 OR “carbonic anhydride” OR “carbon dioxide” OR ozone OR O3 OR O-3 OR smoke OR soot OR diesel OR “black carbon” OR SO2 OR SOx OR “sulfur dioxide” OR “sulfurous anhydride” OR nitrogen OR NO2 OR NO OR NOx OR “nitrogen dioxide” OR “nitrogen oxide*” OR “nitrogen monoxide” OR “volatile organic compound*” OR VOCs OR VOC OR “SVOC” OR “semivolatile organic compound*” OR ((traffic* OR “traffic related”) AND pollut*) OR EMB.EXACT("diesel particulate matter") OR EMB.EXACT("ultrafine particulate matter") OR EMB.EXACT("nitrogen dioxide") OR EMB.EXACT("nitric oxide") OR EMB.EXACT("sulfur dioxide") OR EMB.EXACT("sulfur oxide") OR EMB.EXACT("nitrous oxide emission") OR EMB.EXACT("smoke") OR EMB.EXACT("particulate matter 10") OR EMB.EXACT("exhaust gas") OR EMB.EXACT("indoor air pollution") OR EMB.EXACT("carbon monoxide") OR EMB.EXACT("nitrous oxide") OR EMB.EXACT("carbon dioxide") OR EMB.EXACT("particulate matter 2.5") OR EMB.EXACT("air pollutant") OR EMB.EXACT("air pollution") OR EMB.EXACT.EXPLODE("respirable particulate matter") OR EMB.EXACT.EXPLODE("atmospheric particulate matter") OR EMB.EXACT("nitrogen oxide") OR EMB.EXACT("soot") OR EMB.EXACT("black carbon") OR EMB.EXACT("ozone depletion") OR EMB.EXACT.EXPLODE("air pollution") OR EMB.EXACT("polycyclic aromatic hydrocarbon") OR EMB.EXACT("ozone") OR EMB.EXACT("volatile organic compound"))) AND pd(2000-2021) | 1,411 | Added more geography terms based on CABI descriptors. Mainly provinces and cities in South Africa  Limited to only search Embase database |

Table S2. Assessing risk of publication bias for spirometry (predicted percent) and respiratory infection outcomes using Egger’s, Pustejovsky-Rodgers, and Thompson regressions

| Continuous outcomes | | | | |
| --- | --- | --- | --- | --- |
| Test | Measure | Intercept | t-value | p-value |
| Egger’s | FEV_1_ | -2.48 (1.10) | 2.33 | 0.26 |
|  | FVC | -1.34 (0.76) | 1.79 | 0.32 |
|  | FEV_1_/FVC | -1.33 (0.80) | 1.83 | 0.32 |
|  | PEFR | -2.09 (1.27) | 1.86 | 0.31 |
|  | FEF_25-75_ | -2.50 (0.72) | 3.51 | 0.18 |
| Pustejovsky-  Rodger’s | FEV_1_ | -2.62 (1.42) | 1.91 | 0.31 |
|  | FVC | -1.36 (0.79) | 1.72 | 0.34 |
|  | FEV_1_/FVC | -1.38 (0.91) | 1.67 | 0.34 |
|  | PEFR | -2.32 (1.83) | 1.42 | 0.39 |
|  | FEF_25-75_ | -2.59 (0.92) | 2.86 | 0.21 |
| Thompson | FEV_1_ | -2.48 (1.11) | 2.31 | 0.26 |
|  | FVC | -1.34 (1.18) | 1.15 | 0.46 |
|  | FEV_1_/FVC | -1.33 (1.14) | 1.29 | 0.42 |
|  | PEFR | -2.17 (1.28) | 1.94 | 0.30 |
|  | FEF_25-75_ | -2.50 (1.14) | 2.22 | 0.27 |
| Binary outcomes | | | | |
| Test | Measure | Intercept | t-value | p-value |
| Harbord | Respiratory  Infections | -0.01 (0.09) | -0.58 | 0.62 |
| Peters | Respiratory  Infections | 0.06 (0.08) | -2.22 | 0.11 |

*Note that given the small number of studies present these results should be cautiously interpreted and require more studies to interpret results with confidence

Table S3. Results of the leave one out analysis for meta-analyses

| FEV_1_ | | | | |
| --- | --- | --- | --- | --- |
| Study | SMD (95% CI) | p-value | τ^2^ | I^2^ (%) |
| Omitting Critchley et al. 2015 | 0.14  (-0.93, 0.65) | 0.27 | 0.00 | 0.0 |
| Omitting Dohoo et al. 2012 | 0.24  (-5.48, 5.96) | 0.69 | 0.34 | 83.3 |
| Omitting Oluwole et al. 2012 | 0.31  (-4.62, 5.23) | 0.57 | 0.22 | 74.3 |
| Omitting Wolff et al. 2021 | 0.12  (-1.06, 1.31) | 0.70 | 0.15 | 68.5 |
| FVC | | | | |
| Omitting Critchley et al. 2015 | 0.11  (-0.37, 0.15) | 0.12 | 0.00 | 0.0 |
| Omitting Dohoo et al. 2012 | 0.06  (-2.79, 2.90) | 0.84 | 0.04 | 36.7 |
| Omitting Oluwole et al. 2012 | 0.10  (-2.51, 2.71) | 0.72 | 0.01 | 12.0 |
| FEV_1_/FVC | | | | |
| Omitting Critchley et al. 2015 | 0.02  (-0.25, 0.29) | 0.55 | 0.00 | 0.0 |
| Omitting Dohoo et al. 2012 | 0.22  (-3.00, 3.43) | 0.55 | 0.06 | 49.4 |
| Omitting Oluwole et al. 2012 | 0.26  (-2.71, 3.23) | 0.47 | 0.03 | 31.1 |
| PEFR | | | | |
| Omitting Critchley et al. 2015 | 0.05  (-0.10, 0.20) | 0.14 | 0.00 | 0.0 |
| Omitting Dohoo et al. 2012 | 0.47  (-5.32, 6.26) | 0.49 | 0.35 | 83.2 |
| Omitting Oluwole et al. 2012 | 0.49  (-5.15, 6.14) | 0.47 | 0.32 | 79.8 |
| FEF_25-75_ | | | | |
| Omitting Critchley et al. 2015 | 0.16  (-1.56, 1.24) | 0.39 | 0.00 | 0.0 |
| Omitting Dohoo et al. 2012 | 0.15  (-5.19, 5.48) | 0.79 | 0.29 | 81.1 |
| Omitting Oluwole et al. 2012 | 0.26  (-3.66, 4.19) | 0.55 | 0.11 | 60.1 |
| Respiratory Infections | | | | |
| Study | RR (95% CI) | p-value | τ^2^ | I^2^ (%) |
| Omitting Adane et al. 2021 | 0.81  (0.36, 1.80) | 0.46 | 0.23 | 90.6 |
| Omitting Wafula et al. 2000 | 1.01  (0.79, 1.29) | 0.88 | 0.02 | 57.9 |
| Omitting Jack et al. 2021 | 0.80  (0.36, 1.77) | 0.44 | 0.23 | 90.5 |
| Omitting Kirby et al. 2017 | 0.84  (0.38, 1.88) | 0.55 | 0.24 | 90.4 |
| Omitting Mortimer et al. 2017 | 0.76  (0.38, 1.50) | 0.29 | 0.16 | 88.1 |
